# Discovery of *Caralluma*-derived pregnane glycosides as potent and selective cholinesterase inhibitors: integrated *in silico* and *in vitro* evaluation

**DOI:** 10.1039/d6ra01548d

**Published:** 2026-05-13

**Authors:** Ahmed A. Al-Karmalawy, Mohamed Ibrahim Attia, Radwan Alnajjar, Riham A. El-Shiekh, Arwa Omar Al Khatib, Tarek A. Yousef, Essam Abdel-Sattar

**Affiliations:** a Department of Pharmaceutical Chemistry, Faculty of Pharmacy, Horus University-Egypt New Damietta 34518 Egypt akarmalawy@horus.edu.eg; b College of Science, Chemistry Department, Imam Mohammad Ibn Saud Islamic University (IMSIU) Riyadh 11623 Saudi Arabia tayousef@imamu.edu.sa; c Department of Chemistry, Faculty of Science, University of Benghazi Benghazi Libya; d Pharmacognosy Department, Faculty of Pharmacy, Cairo University Kasr El-Aini Street 11562 Cairo Egypt; e Faculty of Pharmacy, Al-Ahliyya Amman University Amman Jordan

## Abstract

Alzheimer's disease (AD) is the fourth leading cause of death among elderly people worldwide. It has a complex pathogenesis, making multitarget-directed ligands (MTDLs) a key therapeutic strategy. This study evaluated pregnane glycosides isolated from *Caralluma* species (Apocynaceae) as potential cholinesterase inhibitors targeting acetylcholinesterase (AChE) and butyrylcholinesterase (BuChE) enzymes for AD treatment. *In silico* molecular docking against AChE (PDB: 4EY7) and BuChE (PDB: 8CGO) identified caratuberside E and awdelioside B as top AChE binders (−11.09 and −11.49 kcal mol^−1^, outperforming the cocrystal inhibitor at −9.52 kcal mol^−1^). For BuChE, caratuberside G and penicilloside C showed superior scores (−10.94 and −11.55 kcal mol^−1^*vs.* −8.89 kcal mol^−1^ for the cocrystal). These results were validated by 200 ns molecular dynamics simulations (stable RMSD values) and MM-GBSA binding free-energy calculations, confirming strong interactions and favourable energetics. *In vitro* assays (using donepezil as reference) demonstrated potent inhibition: caratuberside E was most active against AChE (IC_50_ = 0.69 ± 0.07 µM), followed by awdelioside B (IC_50_ = 18.99 ± 0.06 µM); caratuberside G (IC_50_ = 1.59 ± 0.16 µM) and penicilloside C (IC_50_ = 12.38 ± 0.51 µM) excelled against BuChE. Collectively, these pregnane glycosides from *Caralluma* show promise as selective cholinesterase inhibitors and potential MTDLs for AD therapy.

## Abstract 2

Discovery of *Caralluma*-derived pregnane glycosides as potent and selective cholinesterase inhibitor.

## Graphical abstract

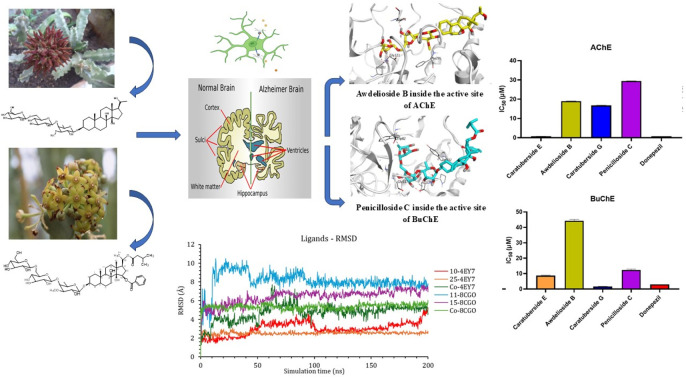

## 1. Introduction

Alzheimer's disease (AD), the most prevalent type of dementia, is a progressive neurodegenerative disorder and ranks as the fourth leading cause of death among older adults globally.^1,2^ The 2021 report from Alzheimer's Disease International (ADI) states that over 55 million individuals globally are affected by AD. The number of cases is expected to increase significantly, reaching 78 million by 2030, highlighting the severe threat that AD poses to global health and the substantial challenges it presents to society.^3^ Currently, treatment for AD primarily focuses on managing symptoms due to its complex etiology, which involves multiple factors, including internal biological processes, genetics, and environmental influences.^4^ Among FDA-approved drugs for AD, aducanumab is the only etiological treatment; however, its effectiveness remains controversial due to its limited success in phase III clinical trials.^5^ While cholinesterase inhibitors do not halt disease progression and show limited effectiveness in advanced AD, they have been proven to significantly enhance cognitive function and remain the primary drugs used in clinical AD treatment.^6,7^ Consequently, the development of new cholinesterase inhibitors, particularly multitarget-directed ones that can simultaneously decrease amyloid plaques, counteract neuroinflammation, combat oxidative stress, support neurons, and provide additional therapeutic effects, is highly important in the fight against AD.^8–14^

The unique characteristics of the chemical entities of natural compounds have resulted in biologically active candidates with promising ADMET profiles (absorption, distribution, metabolism, excretion, and toxicity).^15^ Pregnane glycosides, which are biologically interesting naturally occurring compounds, have been mostly unexplored to date. They are highly important in natural medicine and are well known for their characteristic structural features and significant diverse bioactivities. Pregnane glycosides are distributed in Asclepiadaceae, Apocynaceae, Zygophyllaceae, Ranunculaceae, and Malpighiaceae plants. Researchers have isolated them from several plants and investigated their biological activities, including antibacterial,^16^ cytotoxic,^17^ anti-inflammatory,^18–20^ antiobesity,^21^ anticholinesterases,^18^ and antidiabetic^22^ activities. Pregnane glycosides are a class of compounds with cyclopentane-perhydrophenanthrene as the basic mother nucleus, and 347 compounds have been isolated and identified.^23^

Natural compounds constitute a valuable approach for finding multitarget-directed drug candidates, standing out as an effective strategy for discovering anti-AD agents. The development of multifunctional natural compounds is due to the presence of suitable pharmacophore frameworks for optimal fusion, with target-based pharmacophore identification being particularly promising. Due to their inherent structural features, pregnane glycosides offer multiple advantages, including increased biological activity and efficacy. Consequently, it is regarded as a privileged scaffold in the design of new drugs for various challenging diseases.^24–26^ The neurotransmitter Acetylcholine (ACh), as the patriarch for regulating cognitive and behavioral impairments and neuropsychiatric disturbances, is the direct target of cholinesterase. Considering the extensive interest in pregnane glycosides because of their diverse structures and excellent biological activities, we investigated them as cholinesterase inhibitors. Through these virtual screening experiments, we selected 21 compounds for their potential binding against cholinesterase enzymes, which were validated *in vitro* for their anti-Alzheimer activity as the most promising candidates to accelerate drug development in this area.

## 2. Results and discussion

### *2.1*. *In silico* studies

#### 2.1.1. Molecular docking

The pregnane glycosides isolated from certain *Caralluma* species were subjected to molecular docking studies to investigate their inhibitory potentials against acetylcholinesterase enzyme (AChE) (PDB ID: 4EY7) and butyrylcholinesterase enzyme (BuChE) (PDB ID: 8CGO) (Table 1). The cocrystallized inhibitor of each target was inserted as a reference standard in each docking process. Notably, caratuberside E and awdelioside B were found to be superior candidates for AChE (PDB ID: 4EY7); however, caratuberside G and penicilloside C were described as frontier candidates for BuChE (PDB ID: 8CGO).

**Table 1:** Molecular docking scores of pregnane glycosides isolated from certain *Caralluma* species towards the binding sites of acetylcholinesterase enzyme (AChE) (PDB ID: 4EY7) and butyrylcholinesterase enzyme (BuChE) (PDB ID: 8CGO) compared with those of the cocrystallized inhibitors

No	Compound	Structure	AChE binding score (kcal mol^−1^)	BuChE binding score (kcal mol^−1^)	Ref.
1	Russelioside A	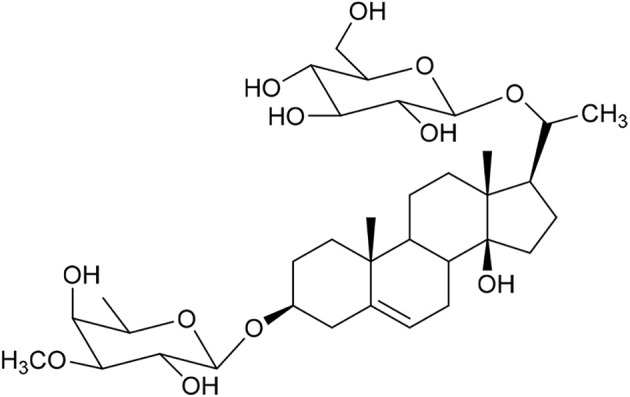	−6.63	−9.89	16 and 27
2	Russelioside B	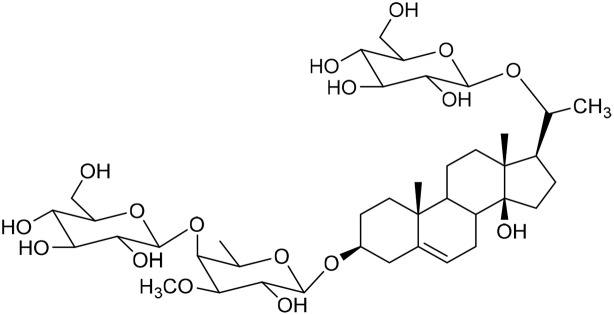	−8.25	−10.40	16 and 27
3	Russelioside C	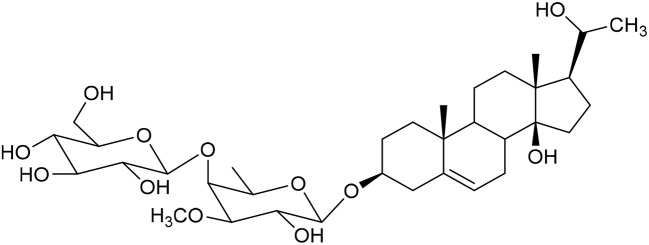	−7.53	−10.32	16 and 27
4	Russelioside D	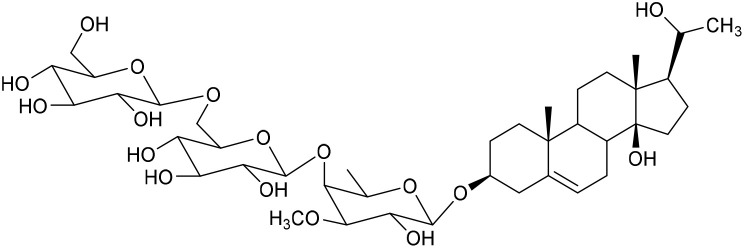	−9.69	−10.04	16 and 27
5	Russelioside E	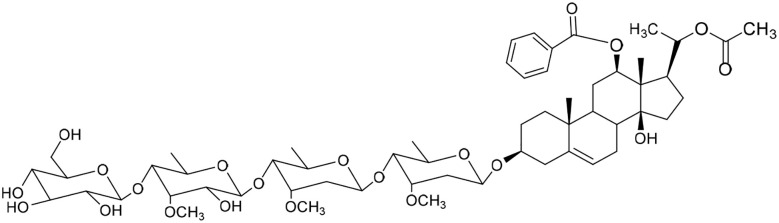	−9.49	—	16, 27 and 28
6	Caratuberside A	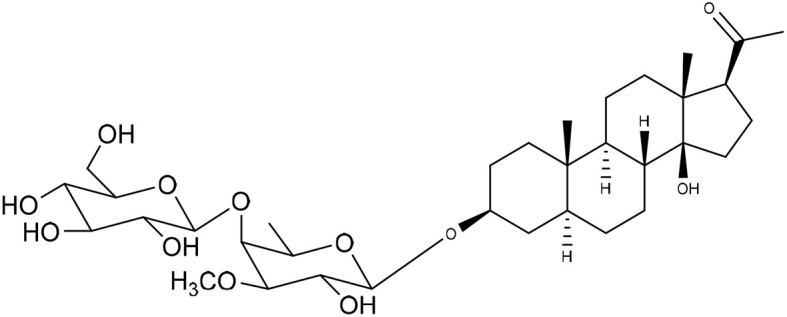	−9.14	−10.47	17
7	Caratuberside B	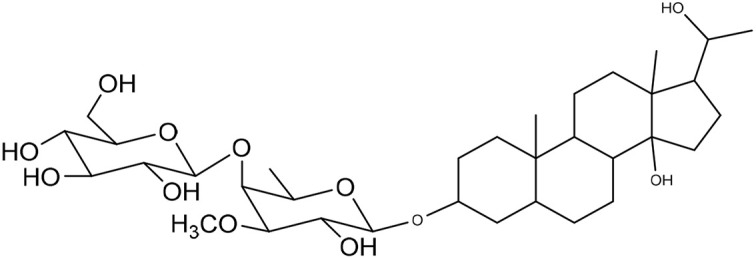	−8.25	−10.08	17
8	Caratuberside C	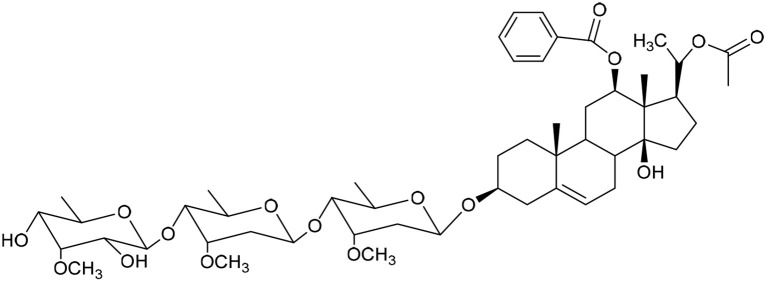	−10.45	—	17
9	Caratuberside D	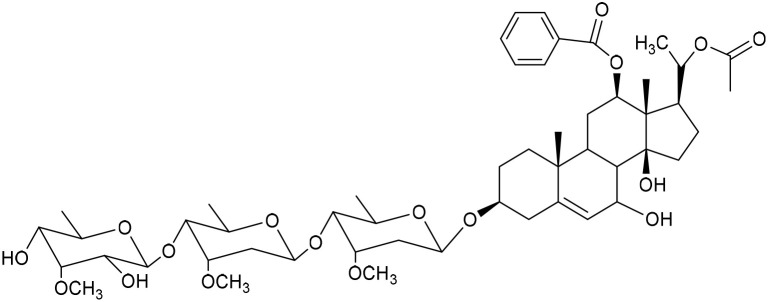	−9.55	—	17
10	Caratuberside E	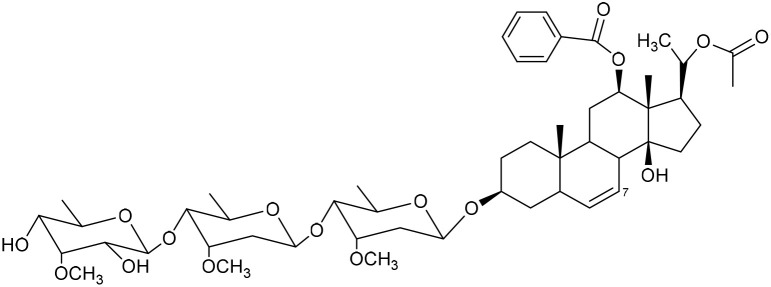	−11.09	−10.10	17
11	Caratuberside G	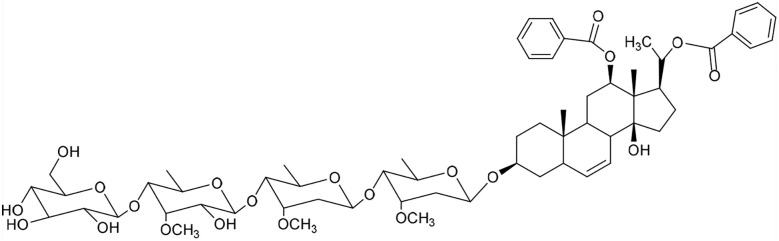	−8.22	−10.94	17
12	Caratuberside F	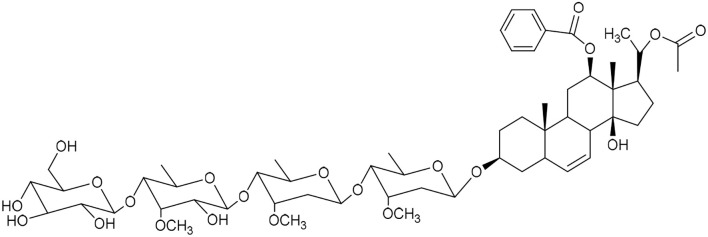	−9.29	—	17
13	Penicilloside A	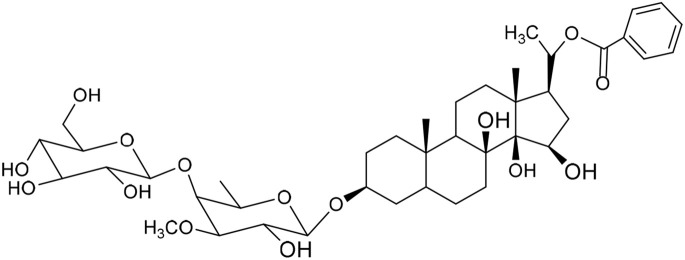	−8.85	−10.22	29 and 30
14	Penicilloside B	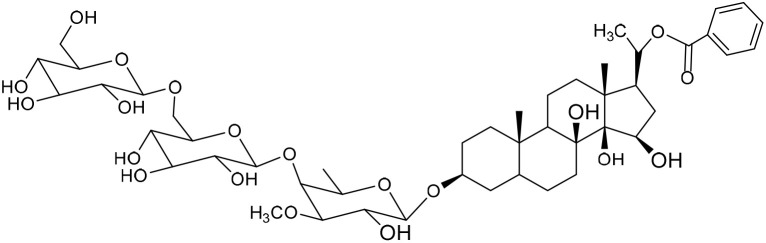	−9.83	—	29 and 30
15	Penicilloside C	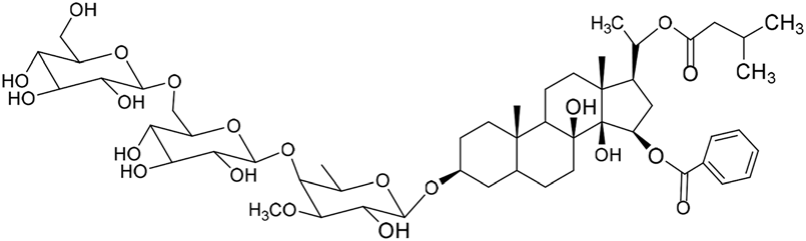	−10.29	−11.55	29 and 30
16	Penicilloside D	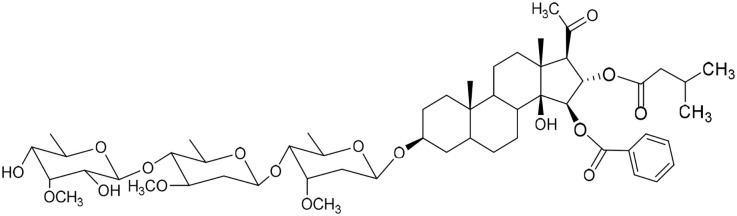	−9.99	—	29 and 30
17	Penicilloside E	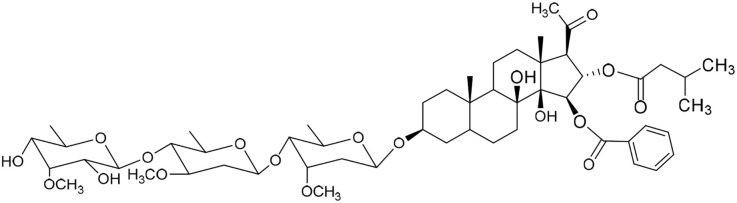	−9.91	−10.51	29 and 30
18	Penicilloside F	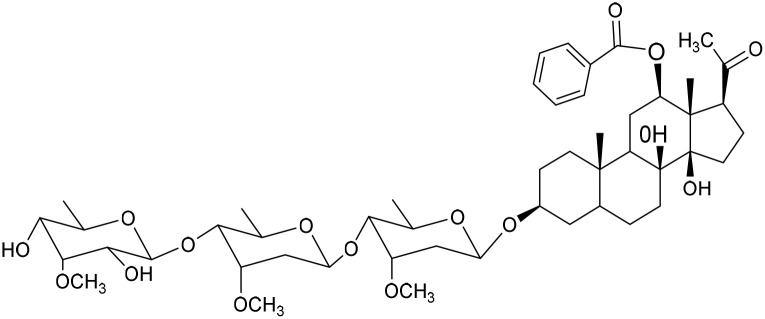	−9.21	−10.39	29 and 30
19	Penicilloside G	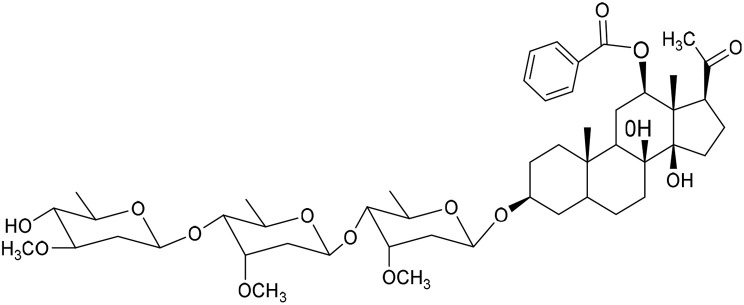	−9.28	−10.33	29 and 30
20	Arabincoside A	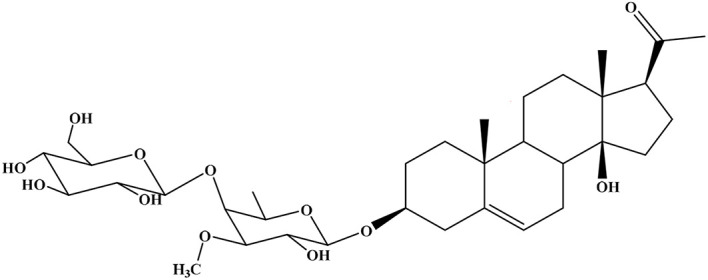	−8.29	−10.13	31
21	Arabincoside B	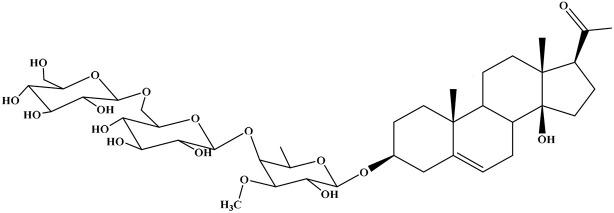	−9.64	−10.60	31
22	Arabincoside C	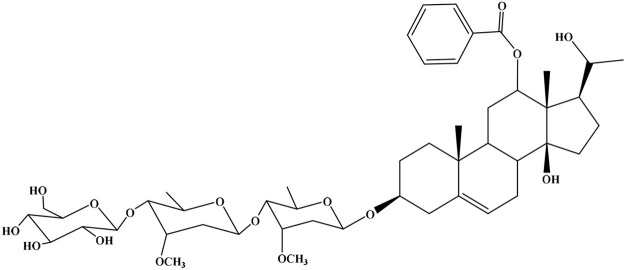	−10.26	−10.87	31
23	Arabincoside D	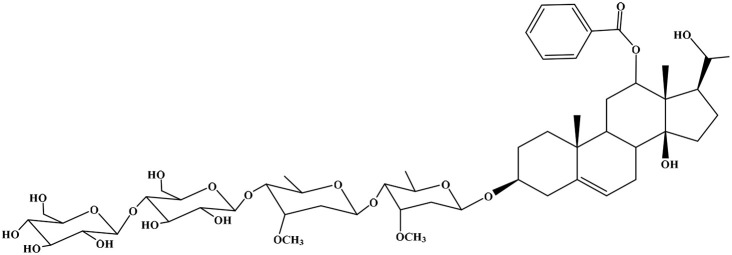	−8.18	—	31
24	Awdelioside A	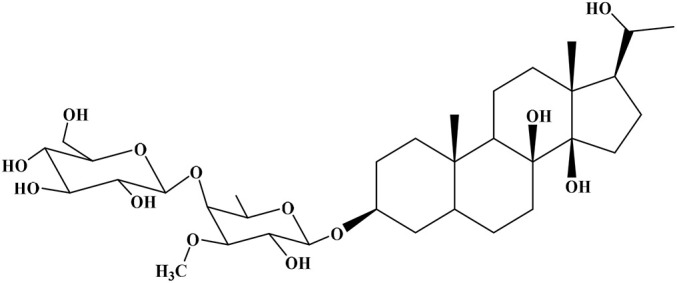	−8.25	−10.36	18
25	Awdelioside B	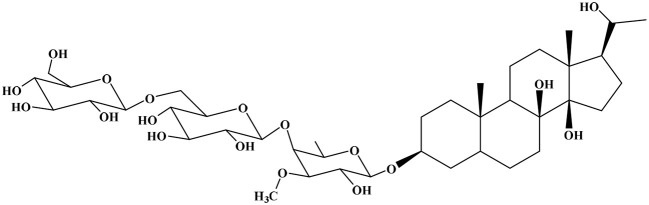	−11.49	−10.51	18
Cocrystal of AChE (donepezil)			−9.52		—
Cocrystal of BuChE				−8.89	—

**Fig. 1:**
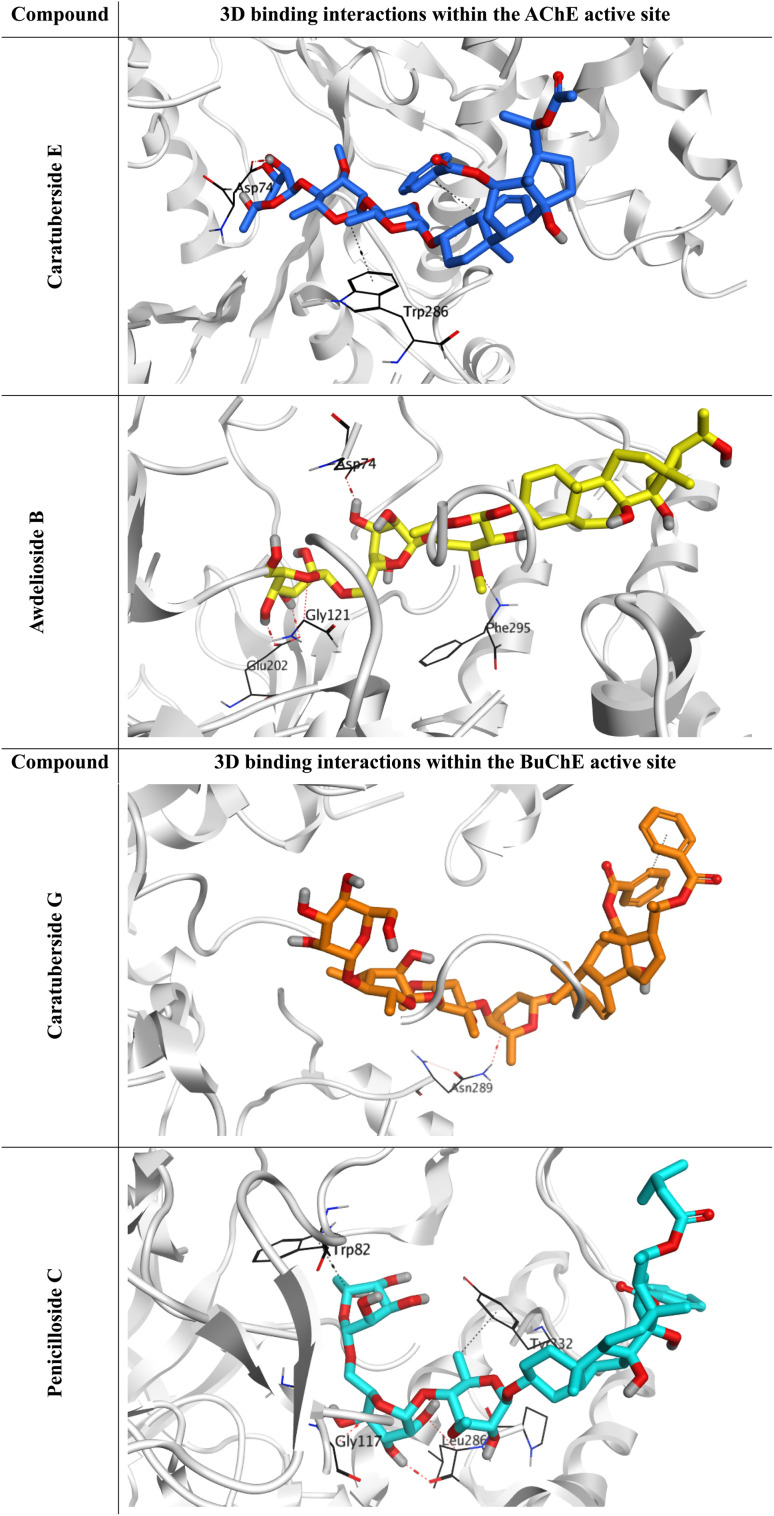
3D binding interactions of caratuberside E and awdelioside B within the active site of AChE (PDB ID: 4EY7), and 3D binding interactions of caratuberside G and penicilloside C within the active site of BuChE (PDB ID: 8CGO).

The docking scores for caratuberside E and awdelioside B (within the AChE active site) were found to be −11.09 and −11.49 kcal mol^−1^ at root mean square deviation (RMSD) values of 1.76 and 1.92 Å, respectively. The cocrystal of AChE had a docking score of −9.52 kcal mol^−1^ (RMSD = 1.98). Moreover, caratuberside E formed one hydrogen bond with Asp74 and one hydrogen–pi bond with Trp286. In addition, awdelioside B (25) has five hydrogen bonds with Asp74, Glu202 (2), Phe295, and Gly121. The cocrystal of AChE presented two hydrogen bonds with Asp74 and Phe295 in addition to one hydrogen–pi bond and one pi–pi bond with Trp286 (Fig. 1).

On the other hand, the docking scores for caratuberside G and penicilloside C (within the BuChE active site) were found to be −10.94 and −11.55 kcal mol^−1^ at RMSD values of 1.58 and 1.67 Å, respectively. The cocrystal of BuChE had a docking score of −8.89 kcal mol^−1^ (RMSD = 1.68). Furthermore, caratuberside G formed one hydrogen bond with Asn289. Additionally, penicilloside C formed three hydrogen bonds (with Leu286 (2) and Gly117) and two hydrogen–pi bonds (with Tyr332 and Trp82). The cocrystal of BuChE presented one hydrogen–pi bond with Tyr332 (Fig. 1).

#### 2.1.2. Structure–activity relationship (SAR)

The superior docking scores and observed selectivity of the top pregnane glycosides can be explained by specific structural features and by differences in the active-site architectures of AChE and BuChE. The rigid pregnane steroidal core facilitates deep penetration into the catalytic gorge, while the glycosidic moieties provide multiple hydroxyl groups for hydrogen bonding. In AChE (PDB: 4EY7), caratuberside E and awdelioside B benefit from strong interactions with the peripheral anionic site (PAS) residues, particularly Asp74, Glu202, and Trp286, as well as the catalytic triad vicinity (Gly121, Phe295). Awdelioside B's five hydrogen bonds with these residues, including multiple contacts with Glu202, likely contribute to its high affinity and AChE preference.

In contrast, BuChE (PDB: 8CGO) possesses a larger acyl pocket due to the replacement of two bulky phenylalanine residues (Phe295 and Phe297 in AChE) with smaller leucine and valine residues. This reduced steric hindrance allows better accommodation of the bulky sugar chains in caratuberside G and penicilloside C, resulting in favorable hydrogen bonds with Asn289, Leu286, Gly117, Glu197, and π-interactions with Trp82 and Tyr332. Consequently, caratuberside E shows clear AChE selectivity, while caratuberside G and penicilloside C exhibit stronger BuChE inhibition. These differences highlight how the glycosidic nature and hydroxylation pattern of pregnane glycosides can be tuned for selective targeting of either enzyme, offering opportunities for designing dual or selective inhibitors for Alzheimer's therapy.

Collectively, the aforementioned results strongly suggest the inhibitory potential of pregnane glycosides isolated from certain *Caralluma* species, (especially caratuberside E and awdelioside B (against AChE) and caratuberside G and penicilloside C (against BuChE)). These candidates surpassed the docking scores of the cocrystallized inhibitors and described comparable binding modes as well.

#### 2.1.3. Molecular dynamics simulation

**Fig. 2:**
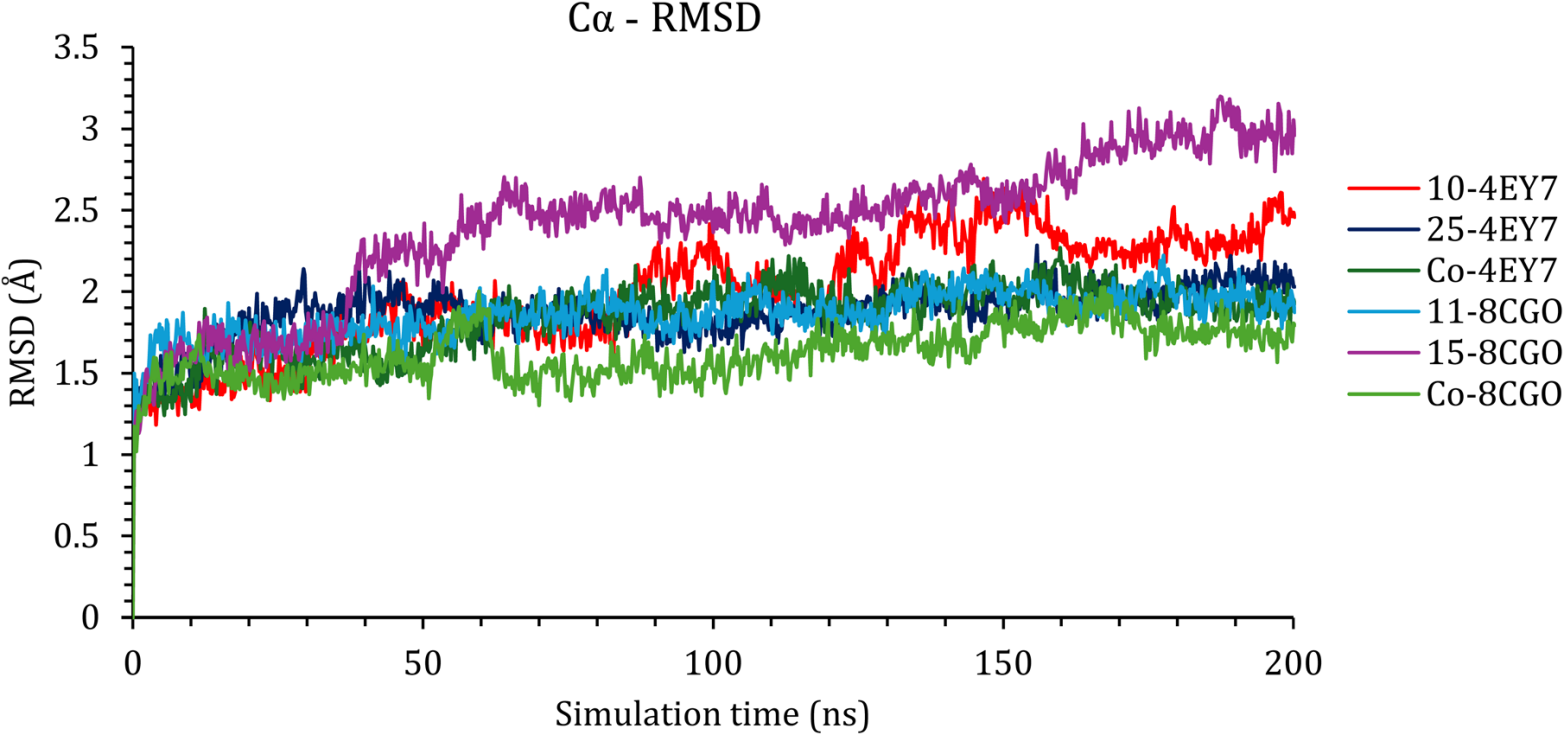
The RMSD of the C_α_ of the protein backbone within the complexes as a function of simulation time.

In an attempt to validate the docking results, molecular dynamics was implemented for 200 ns for the best two compounds in each protein. First, the RMSD for the Cα atoms in each protein was monitored and plotted as a function of simulation time (Fig. 2).

As shown in Fig. 2, the RMSDs of the C_α_ for all the complexes were within acceptable ranges of less than 3.00 Å. The C_α_ of the AChE enzyme (PDB ID: 4EY7) was stable, with caratuberside E showing an RMSD of 2.00 Å until approximately 100 ns; then, it started to fluctuate until it reached 2.50 Å at approximately 120 ns, which was at the end of the simulation. Awdelioside B was also stable and had an RMSD of 1.50 Å toward the end of the simulation time.

In the case of the BuChE enzyme, caratuberside G did not affect the stability of the conformation of the protein, and the Cα RMSD was approximately 1.50–2.00 Å. In contrast, penicilloside C had little effect on the conformation of the protein, and the RMSD was greater than that of caratuberside G, which was 3.00 Å; however, this RMSD was within the acceptable range.

**Fig. 3:**
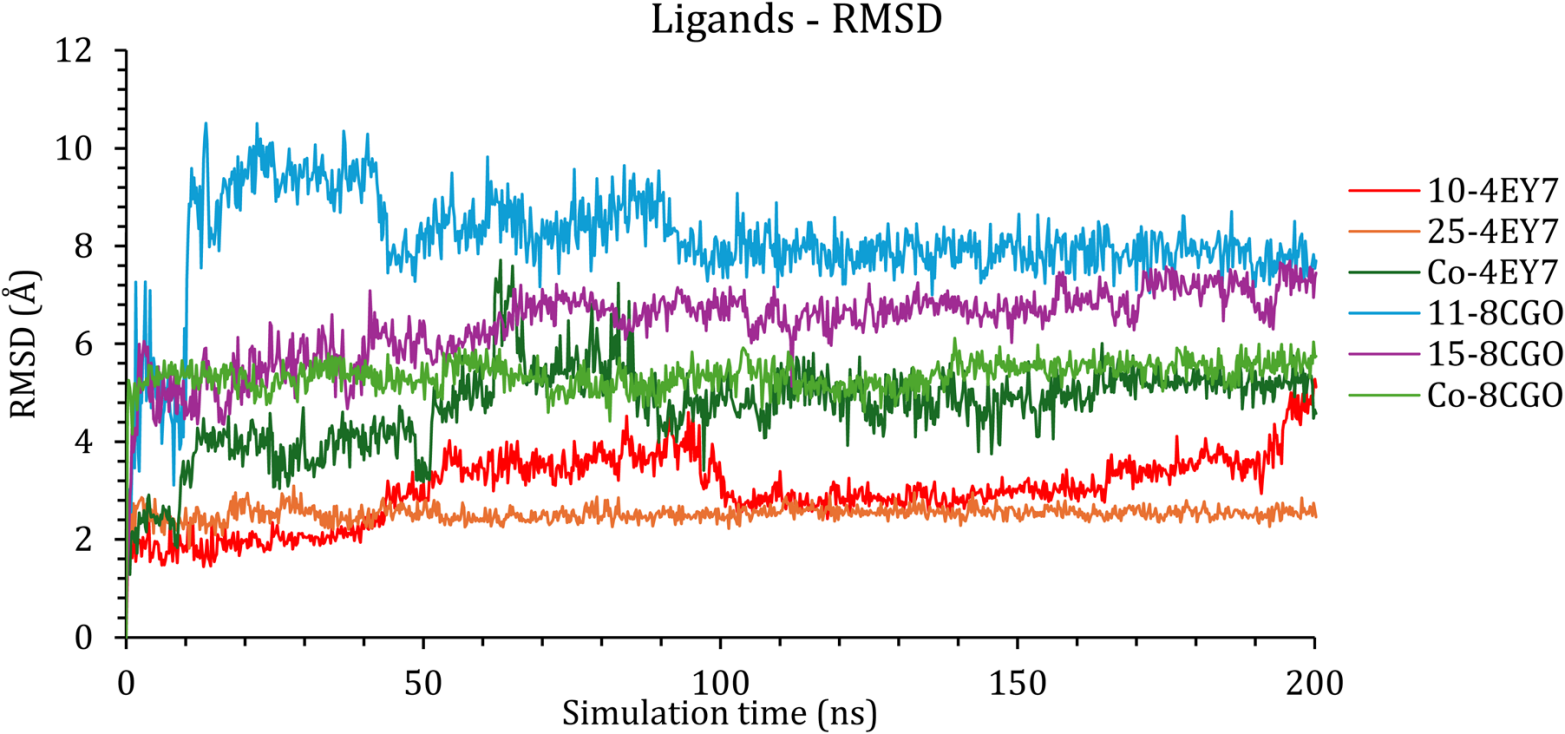
The RMSD of the ligands inside the active site of the protein with respect to their initial position as a function of simulation time.

Next, the RMSDs of the frontrunner compounds inside the active sites of AChE and BuChE were monitored and plotted as a function of time (Fig. 3).

**Fig. 4:**
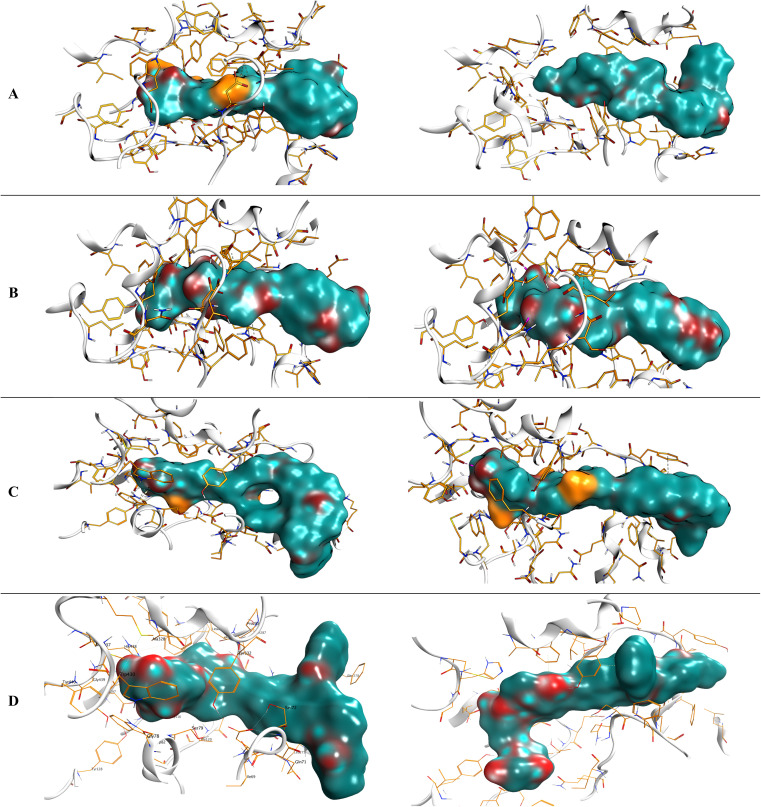
Structures of the ligands inside the active site of the targeted protein left (0 ns) and right (200 ns) for caratuberside E (A), awdelioside B (B), caratuberside G (C), and penicilloside C (D).

Caratuberside E was highly stable inside the active site of the AChE protein and presented an RMSD of less than 4.00 Å from the beginning until approximately 180 ns, where its fluctuation started to increase gradually (Fig. 4a). Awdelioside B was even more stable than caratuberside E and the cocrystal ligand, with an RMSD of 2.50 Å throughout the simulation time (Fig. 4b).

In the case of BuChE, caratuberside G showed an RMSD of approximately 10.00 Å, which is quite high; however, upon examining the behavior of the compound inside the active site, as shown in Fig. 4c, the docked pose revealed that caratuberside G forms an intramolecular H-bond and forms a cyclic structure that opens to the acyclic form at approximately 10 ns of simulation time. Once the acyclic form is established, it remains throughout the simulation. Finally, penicilloside C moved by an RMSD of 6.00–7.00 Å from the beginning of the simulation, which reflects a significant conformational adaptation of the binding pocket rather than instability. Penicilloside C reoriented itself inside the active site, as shown in Fig. 4d.

**Fig. 5:**
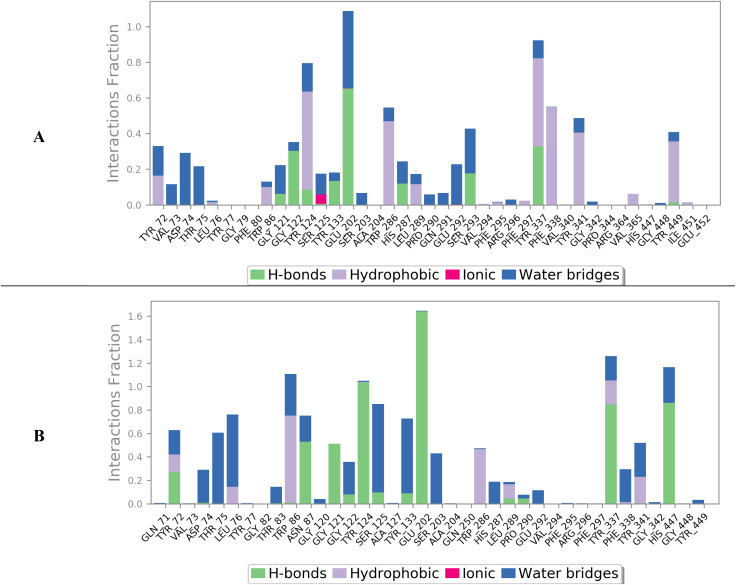
Interactions between AChE amino acid residues and caratuberside E (A) and awdelioside B (B).

Next, the protein–ligand contacts were analysed at the amino acid residue level. Caratuberside E showed a strong H-bond interaction with Glu202, along with weaker H-bonds with Gly122 and Tyr337 (Fig. 5a). Awdelioside B showed almost 180% interaction with Glu202, which indicates that more than one H-bond formed during the simulation. A strong H-bond was formed with residues Tyr124 (110%), Try337 (95%), and His447 (95%), along with weaker residues such as Asn87 (60%) and Gly121 (55%) (Fig. 5b).

**Fig. 6:**
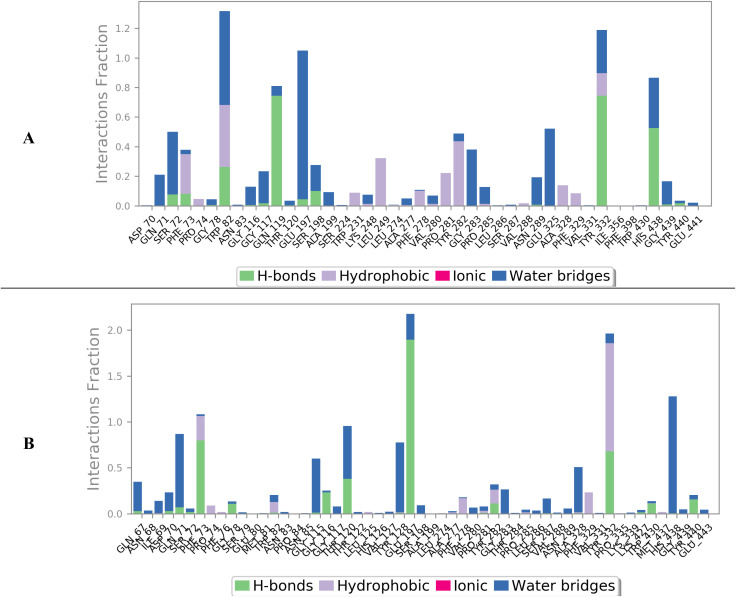
Interactions between BuChE amino acid residues and caratuberside G (A) and Penicilloside C (B).

In the BuChE case, caratuberside G was able to form three H-bond interactions with the amino acids Gln119 (80%), Try332 (80%), and His438 (55%), along with multiple hydrophobic interactions (Fig. 6a). Penicilloside C, on the other hand, was able to form a strong H-bond interaction with residue Glu197 (190%), in addition to Phe73 (90%) and Try332 (70%) (Fig. 6b).

#### 2.1.4. MM-GBSA binding energies

The thermal_mmgbsa.py Python script of Schrodinger was used to calculate the average MM-GBSA binding energy. Therefore, the Coulomb, covalent binding, hydrogen bonding, lipophilic, generalized Born electrostatic solvation, and van der Waals energies were also calculated. All the results are presented in detail in Table 2.

**Table 2:** Prime MM-GBSA energies for the complexes are reported in kcal mol^−1^^a^

Complex	Δ*G* bind	Coulomb	Covalent	Hbond	Lipo	Packing	Solv_GB	VdW
**AChE (PDB ID: 4EY7)**								
Caratuberside E	−53.52	−6.59	3.90	−0.53	−23.78	−0.10	25.54	−51.96
Awdelioside B	−92.22	−35.10	3.76	−3.58	−33.64	0.00	45.26	−68.92
Cocrystal	−54.20	−18.01	1.39	−0.19	−24.43	−1.84	31.33	−42.43

**BuChE (PDB ID: 8CGO)**								
Caratuberside G	−78.28	−15.00	6.98	−1.27	−29.97	−0.83	32.53	−70.71
Penicilloside C	−56.48	−18.06	4.81	−2.33	−21.58	−0.72	33.61	−52.20
Cocrystal	−64.15	−18.44	1.82	−0.39	−33.91	−6.08	46.50	−53.65

a Coulomb: coulomb energy; covalent: covalent binding energy; H-bond: hydrogen-bonding energy; Lipo: Lipophilic energy; Solv_GB: Generalized Born electrostatic solvation energy; VdW: van der Waals energy.

As shown in Table 2, awdelioside B presented a binding energy of −92.22 kcal mol^−1^, which was superior to that of the cocrystal of AChE (−54.20 kcal mol^−1^), which presented a binding energy of −54.20 kcal mol^−1^. Moreover, caratuberside E presented a binding energy that was close to the cocrystal binding energy.

In the case of BuChE, caratuberside G presented a higher binding energy of −78.28 kcal mol^−1^ than did the cocrystal ligand (−56.48 kcal mol^−1^). On the other hand, penicilloside C presented a lower binding energy of −56.48 kcal mol^−1^.

#### 2.1.5. Absorption, distribution, metabolism, excretion, and toxicity (ADMET) studies

Table 3 clarifies that caratuberside E has better Caco-2 permeability and intestinal absorption (>60%), indicating possible oral administration. The intestinal absorption of caratuberside G is also good (>40%). The volume of distribution for caratuberside E was medium (log VDss = 1.097) and superior to that of caratuberside G (log VDss = 0.422). The CNS permeability of all candidates is low, and so they will need a drug delivery system to help penetrate the BBB. Additionally, all members are Cytochrome P450 inhibitors. Moreover, all analogues have no AMES toxicity, indicating that they are not mutagenic and therefore do not act as carcinogens. Both awdelioside B and penicilloside C are not hepatotoxic. Furthermore, the examined candidates are not skin sensitizers or *h*ERG I inhibitors.

**Table 3:** ADMET properties of caratuberside E, awdelioside B, caratuberside G, and Penicilloside C

		Caratuberside E	Awdelioside B	Caratuberside G	Penicilloside C
Absorption	Caco-2 permeability	0.586	−0.6	−0.437	−0.68
	Intestinal absorption	63.384	0	41.916	0.483
	Water solubility (log *S*)	−3.13	−2.085	−2.888	−2.861
	P-glycoprotein substrate	Yes	Yes	Yes	Yes
	P-glycoprotein I inhibitor	Yes	No	Yes	No
	P-glycoprotein II inhibitor	No	No	No	No
	Skin permeability	−2.735	−2.735	−2.735	−2.735
Distribution	VDss (Human)	1.097	−0.154	0.422	−0.183
	BBB permeability	−2.504	−2.13	−2.934	−2.339
	Fraction unbound (Human)	0.184	0.458	0.279	0.364
	CNS permeability	−3.97	−6.699	−4.762	−5.254
Metabolism	Cytochrome P450 inhibitors	No	No	No	No
Excretion	Renal OCT2 substrate	No	No	No	No
	Total clearance (CLtot)	0.441	0.915	0.294	0.417
Toxicity	*T. pyriformis* toxicity	0.285	0.285	0.285	0.285
	AMES toxicity	No	No	No	No
	Minnow toxicity	5.447	10.595	4.174	7.078
	Maximum tolerated dose	−0.868	−1.037	−0.472	−0.379
	Hepatotoxicity	Yes	No	Yes	No
	Oral rat acute toxicity (LD_50_)	4.673	2.852	3.055	2.535
	Oral rat chronic toxicity (LOAEL)	2.271	4.371	3.547	5.196
	Skin sensitization	No	No	No	No
	*h*ERG I inhibitor	No	No	No	No
	*h*ERG II inhibitor	Yes	Yes	Yes	Yes

### 2.2. Biological evaluation

#### 2.2.1. Acetylcholine esterase (AChE) and butyrylcholine esterase (BuChE) inhibition assays

**Fig. 7:**
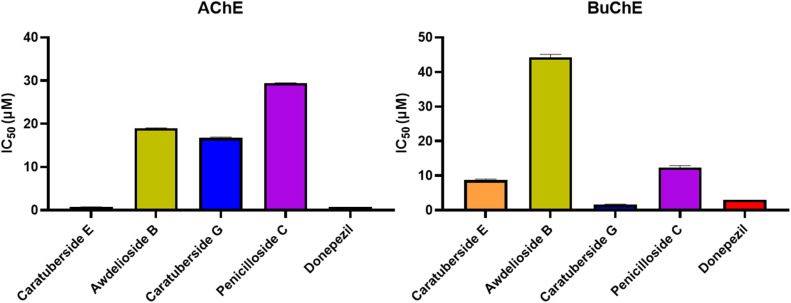
*In vitro* acetylcholine esterase (AChE) and butyrylcholine esterase (BuChE) enzymes inhibition assays of the most active pregnane glycosides against donepezil.

The cholinesterase inhibitory activities of the four tested compounds, caratuberside E, awdelioside B, caratuberside G, and penicilloside C, demonstrated promising efficacy, as reflected by their IC_50_ values (Fig. 7). All the compounds exhibited inhibitory activity against both AChE and BuChE enzymes. Caratuberside E had the strongest inhibitory effect on AChE (IC_50_ = 0.69 µM). Awdelioside B had moderate inhibitory effects on both enzymes (AChE: IC_50_ = 18.99 µM; BuChE: IC_50_ = 44.22 µM). Caratuberside G also demonstrated moderate activity toward both enzymes (AChE: IC_50_ = 29.40 µM; BuChE: IC_50_ = 12.38 µM), whereas penicilloside C exhibited the most potent inhibition against BuChE (IC_50_ = 1.59 µM). This is particularly relevant for Alzheimer's disease therapy, which could be beneficial for targeting cognitive symptoms in neurodegenerative diseases, in addition to their selective cholinesterase inhibition, which could reduce unwanted peripheral side effects. All compounds exhibited dual inhibition against both AChE and BuChE enzymes, outperforming or matching several benchmark natural inhibitors reported in the literature (*e.g.*, galantamine: AChE IC_50_ = 0.31–3.52 µM, BuChE IC_50_ = 5.97–16.2 µM;^32–35^ huperzine A: AChE IC_50_ = 0.07–0.82 µM, BuChE IC_50_ 20–40 µM^36–38^). Where, caratuberside E displayed the strongest AChE inhibition, comparable to huperzine A, and penicilloside C was the most potent BuChE inhibitor, exceeding galantamine and approaching physostigmine (BuChE IC_50_ 0.02–1 µM)^39,40^ with high selectivity. Collectively, these comparisons suggest that pregnane glycosides from *Caralluma* represent a structurally distinct, non-alkaloidal scaffold with cholinesterase inhibitory activity comparable to several benchmark natural products, highlighting their potential as pharmacologically relevant leads.

## 3. Conclusions

We investigated several pregnane glycosides isolated from certain *Caralluma* species by our group for their choline esterase inhibitory activity as valid therapeutic agents for the treatment of Alzheimer's disease, as well as for the use of receptor-based virtual screening to discover biologically active compounds. First, molecular docking, MD simulations, and MM-GBSA calculations revealed that caratuberside E and awdelioside B are superior candidates for AChE; however, caratuberside G and penicilloside C were described as frontier candidates for BuChE compared with the corresponding cocrystal inhibitors. The strategy of virtual screening allowed us to identify caratuberside E as a lead compound in the pursuit of a new AchE lead compound inhibitor with IC_50_ = 0.69 ± 0.07 µM and SI=IC_50BuChE_/IC_50AChE_ = 12.38. Additionally, caratuberside G is a new lead compound in the pursuit of new BuChE lead compound inhibitor with an IC_50_ = 1.59 ± 0.16 µM and an SI=IC_50AChE_/IC_50BuChE_ = 10.51. The structural features identified—particularly the pregnane core combined with specific glycosidic substitutions underpin the observed AChE/BuChE selectivity and provide a rational basis for future lead optimization.

In contrast to classical alkaloidal cholinesterase inhibitors such as galantamine, huperzine A, and physostigmine, the active *Caralluma*-derived pregnane glycosides feature a rigid steroidal core linked to bulky glycosidic chains, representing a distinct chemotype for dual AChE/BuChE modulation. Their comparable *in vitro* potency to these established natural products, combined with their underexplored structural framework, highlights the potential of *Caralluma* pregnane glycosides as promising leads for further investigation in AD therapy.

## 4. Study limitations and future work

While this study provides robust *in silico* and *in vitro* enzyme inhibition data, further investigations, including cell-based neuroprotection assays (*e.g.*, against Aβ-induced toxicity), BBB permeability studies, and *in vivo* AD models, are warranted to fully establish therapeutic potential. The identified pregnane glycosides offer a novel scaffold with potent and selective cholinesterase inhibition, complementing existing natural product leads and providing new opportunities for structural optimization in AD drug discovery.

## 5. Materials and methods

### 5.1. Isolation of pregnane glycosides

A series of 25 pregnane glycosides, previously isolated from various *Caralluma* species (detailed in Table 1), were evaluated *via* molecular docking for their binding affinities toward the active sites of AChE (PDB ID: 4EY7) and BuChE (PDB ID: 8CGO). Among these, four specific candidates—caratuberside E, awdelioside B, caratuberside G, and penicilloside C, which were previously isolated by our group from *C. russelliana*, *C. tuberculata*, *C. penicillata*, and *C. arabica*, demonstrated promising efficacy in subsequent cholinesterase inhibitory assays (SI Fig. S1–S8).

### 5.2. *In silico* studies

#### 5.2.1. Molecular docking

Pregnane glycosides isolated from certain *Caralluma* species were subjected to molecular docking studies to investigate their inhibitory potentials against AChE (PDB ID: 4EY7) and BuChE (PDB ID: 8CGO). The molecular docking process was performed *via* AutoDock Vina,^41^ and visualization was performed *via* PyMOL software.^42^ The chemical structures of the isolated candidates were sketched in ChemDraw and prepared by energy minimization and partial charge optimization.^43^ The target receptors were prepared by hydrogenation (3D), energy minimization, and correction.^44^ Two docking processes were applied, and the most active members of each enzyme were selected for further investigation. Additionally, two validation procedures were performed by redocking the ligand of each receptor within its active site.^45^ The validity of this method was confirmed by similar binding modes (SI Fig. S9) and low RMSD values (0.13 Å for AChE and 1.32 Å for BuChE).^46^

#### 5.2.2. Molecular dynamics simulation

Molecular dynamics simulations over 200 ns were performed for the selected complexes (caratuberside E and awdelioside B) with AChE and (caratuberside G and penicilloside C) with BuChE, which yielded the highest binding scores using the Desmond package of Schrödinger LLC.^47,48^ The method is described in the SI Data (SI1). Moreover, a proof-of-concept study was conducted on caratuberside E, in which the complex MD was replicated in triplicate to validate the MD output (SI Fig. S10 and S11).

#### 5.2.3. MM-GBSA calculations

The molecular mechanics generalized born surface area (MM-GBSA) energies were evaluated *via* the thermal_mmgbsa.py Python script of Schrödinger LLC.^47^ The method is represented in the SI Data (SI2).

#### 5.2.4. ADMET studies

Based on the chemical structures of the examined candidates (caratuberside E, awdelioside B, caratuberside G, and penicilloside C), the pkCSM web platform (https://biosig.lab.uq.edu.au/pkcsm/prediction_single/toxicity_1765271299.12) was used to estimate the ADMET features *in silico*.^49^

### 5.3. Acetylcholine esterase (AChE) and butyrylcholine esterase (BuChE) enzyme inhibition assays

The assays were conducted in a 96-well plate, as reported previously.^50–52^ A microplate reader (Tecan, USA) was used. Bovine serum albumin was purchased from Sigma-Aldrich (St. Louis, MO, USA). AChE from electric eel (type VI-S lyophilized powder, EC 3.1.1.7) and horse BuChE (EC 3.1.1.8) enzymes were purchased from Sigma-Aldrich (St. Louis, MO, USA). Butyrylthiocholine iodide and acetylthiocholine were utilized as substrates in the BuChE and AChE assays, respectively, and were purchased from Sigma-Aldrich (St. Louis, MO, USA). Dithio-bis(2-nitrobenzoic acid) (DTNB) served as an indicator and was purchased from Sigma-Aldrich (St. Louis, MO, USA). The buffers and other chemicals used were of extra pure analytical grade. Briefly, 170 µL of tris–HCl buffer (200 mM, pH 7.5) was added, followed by 20 µL of different concentrations of the tested compounds (10–0.15625 µg mL^−1^) and then 20 µL of the enzyme mixture (0.1 U mL^−1^). After an incubation period of 10 min at 25 °C, 40 µL of DTNB and then 20 µL of the substrate (1.11 mM) were added. All the samples were dissolved in DMSO. The intensity of the developed color was measured at 405 nm *via* a microplate reader (reading A), and the control without the inhibitor was measured (reading B). Blank assays were performed by replacing the enzyme (20 µL) with buffer, and their absorbances were recorded to correct for spontaneous lysis of the indicator or the inherent color of the inhibitor. All the reactions were performed in triplicate. Linear regression was performed to calculate the IC_50_ (50% inhibitory concentration). Microsoft Excel 2010 (Redmond, WA, USA) was used for the data analysis, where the % of inhibition was calculated according to the following equation.
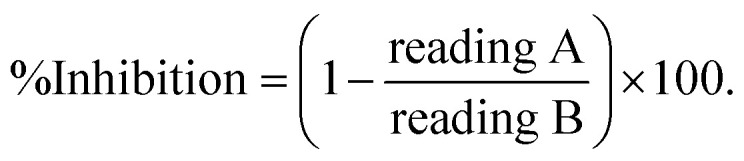


### 5.4. Purity and statistical analysis

All tested compounds were isolated with purity >95% as confirmed by NMR spectroscopy (SI Data). All biological assays were performed in triplicate (*n* = 3 independent experiments), and results are reported as mean ± SD.

## Author contributions

Conceptualization, AAAK, RAES and EAS; methodology, AAAK, MIA, RAES, RA, RAES, and TAY; investigation, AAAK, MIA, RAES, RA, RAES, and TAY; formal analysis, AAAK, MIA, RAES, RA, RAES, AOAK, and TAY; writing—original draft preparation, AAAK, MIA, RAES, RA, RAES, TAY, and EAS; writing—review and editing, AAAK, MIA, RAES, RA, RAES, AOAK, TAY, and EAS; visualization, AAAK, MIA, RAES, RA, RAES, and TAY; supervision, EAS and AAAK.

## Conflicts of interest

I declare that the authors have no competing interests as defined, or other interests that might be perceived to influence the results and/or discussion reported in this paper.

## Supplementary Material

RA-016-D6RA01548D-s001

## Data Availability

The data supporting this article have been included in the manuscript and the supplementary information (SI) file. Supplementary information is available. See DOI: https://doi.org/10.1039/d6ra01548d.

## References

[cit1] Ashraf G. M., Tarasov V. V., Makhmutova A., Chubarev V. N., Avila-Rodriguez M., Bachurin S. O., Aliev G. (2019). Mol. Neurobiol..

[cit2] Leng F., Edison P. (2021). Nat. Rev. Neurol..

[cit3] Gauthier S. , Rosa-Neto P., Morais J. and Webster C., World Alzheimer report 2021: Journey through the Diagnosis of Dementia, Alzheimer's Disease International, London, 2022

[cit4] Shao W., Peng D., Wang X. (2017). J. Clin. Neurosci..

[cit5] Behl T., Kaur I., Sehgal A., Singh S., Sharma N., Makeen H. A., Albratty M., Alhazmi H. A., Felemban S. G., Alsubayiel A. M. (2022). Biomed. Pharmacother..

[cit6] Atri A. (2019). Med. Clin..

[cit7] Ju Y., Tam K. Y. (2022). Neural Regen. Res..

[cit8] Travers-Lesage V., Mignani S. M., Dallemagne P., Rochais C. (2022). Expert Opin. Drug Discov..

[cit9] Ibrahim M. M., Gabr M. T. (2019). Neural Regen. Res..

[cit10] Wang N., Qiu P., Cui W., Yan X., Zhang B., He S. (2019). Curr. Med. Chem..

[cit11] Gontijo V. S., Viegas F. P. D., Ortiz C. J., de Freitas Silva M., Damasio C. M., Rosa M. C., Campos T. G., Couto D. S., Tranches Dias K. S., Viegas C. (2020). Curr. Neuropharmacol..

[cit12] Athar T., Al Balushi K., Khan S. A. (2021). Mol. Biol. Rep..

[cit13] Al-Karmalawy A. A. , Mohamed A. F., Shalaby H. N., Elmaaty A. A., El-Shiekh R. A., Zeidan M. A., Alnajjar R., Alzahrani A. Y. A., Al Mughram M. H., Shaldam M. A. and Tawfik H. O., RSC Medicinal Chemistry, 2025, 10.1039/D4MD00778F PMC11865952 40027342

[cit14] Eissa K. I., Kamel M. M., Mohamed L. W., Doghish A. S., Alnajjar R., Al-Karmalawy A. A., Kassab A. E. (2023). Drug Dev. Res..

[cit15] Katiyar C., Gupta A., Kanjilal S., Katiyar S. (2012). Ayu.

[cit16] El-Shiekh R. A., Hassan M., Hashem R. A., Abdel-Sattar E. (2021). Antibiotics.

[cit17] Abdel-Sattar E., Harraz F. M., Al-Ansari S. M. A., El-Mekkawy S., Ichino C., Kiyohara H., Ishiyama A., Otoguro K., Omura S., Yamada H. (2008). Phytochemistry.

[cit18] El-Shiekh R. A., Shalabi A. A., Al-Hawshabi O. S., Salkini M. A., Abdel-Sattar E. (2023). Steroids.

[cit19] El-Shiekh R. A., Nabil G., Shokry A. A., Ahmed Y. H., Al-Hawshabi O. S., Abdel-Sattar E. (2023). Inflammopharmacology.

[cit20] El-Shiekh R. A., Salama A., Al-Mokaddem A. K., Bader A., Abdel-Sattar E. A. (2021). Steroids.

[cit21] Abdel-Sattar E., Mehanna E. T., El-Ghaiesh S. H., Mohammad H. M., Elgendy H. A., Zaitone S. A. (2018). Front. Pharmacol..

[cit22] Abdel-Sattar E., El-Maraghy S. A., El-Dine R. S., Rizk S. M. (2016). Chem.-Biol. Interact..

[cit23] Si Y., Sha X.-S., Shi L.-L., Wei H.-Y., Jin Y.-X., Ma G.-X., Zhang J. (2022). Phytochem. Lett..

[cit24] Matošević A., Bosak A. (2020). Arh. Hig. Rada. Toksikol..

[cit25] Yılmaz S., Akbaba Y., Özgeriş B., Köse L. P., Göksu S., Gülçin İ., Alwasel S. H., Supuran C. T. (2016). J. Enzym. Inhib. Med. Chem..

[cit26] Ghosh A. K., Brindisi M. (2015). J. Med. Chem..

[cit27] Abdul-Aziz Al-Yahya M., Abdel-Sattar E., Guittet E. (2000). J. Nat. Prod..

[cit28] Abdel-Sattar E., Ahmed A. A., Hegazy M.-E. F., Farag M. A., Al-Yahya M. A.-A. (2007). Phytochemistry.

[cit29] Abdel-Sattar E., Al-Yahya M. A.-A., Nakamura N., Hattori M. (2001). Phytochemistry.

[cit30] Abdel-Sattar E., Meselhy M. R., Al-Yahya M. A.-A. (2002). Planta Med..

[cit31] Abdel-Sattar E. A., Al-Hawshabi O. S., Shalabi A. A., El Halawany A. M., Meselhy M. R. (2022). Tetrahedron.

[cit32] Howes M.-J. R., Houghton P. J. (2009). Int. J. Pharma Bio Sci..

[cit33] Sramek J. J., Frackiewicz E. J., Cutler N. R. (2000). Expert Opin. Invest. Drugs.

[cit34] Satheeshkumar N., Mukherjee P. K., Bhadra S., Saha B. (2010). Phytomedicine.

[cit35] Stavrakov G., Philipova I., Lukarski A., Atanasova M., Zheleva D., Zhivkova Z. D., Ivanov S., Atanasova T., Konstantinov S., Doytchinova I. (2020). Molecules.

[cit36] Suvaiv, Singh K., Hasan S. M., Kumar A., khan A., Shahanawaz M., Zaidi S. M. H., Verma K. (2025). Beni-Suef Univ. J. Basic Appl. Sci..

[cit37] Zaidi H. and Verma K., 2025

[cit38] Xi-Can T., Kindel G. H., Kozikowski A. P., Hanin I. (1994). J. Ethnopharmacol..

[cit39] Atack J. R., Yu Q.-S., Soncrant T. T., Brossi A., Rapoport S. I. (1989). J. Pharmacol. Exp. Ther..

[cit40] Bartolini M., Greig N. H., Yu Q.-s., Andrisano V. (2009). J. Chromatogr. A.

[cit41] Huey R. , Morris G. M. and Forli S., The Scripps Research Institute Molecular Graphics Laboratory, 2012, vol. 10550, p. 1000

[cit42] Yuan S., Chan H. S., Hu Z. (2017). Wiley Interdiscip. Rev.: Comput. Mol. Sci..

[cit43] Belal A., Abdel Gawad N. M., Mehany A. B. M., Abourehab M. A. S., Elkady H., Al-Karmalawy A. A., Ismael A. S. (2022). J. Enzyme Inhib. Med. Chem..

[cit44] Hammouda M. M., Elmaaty A. A., Nafie M. S., Abdel-Motaal M., Mohamed N. S., Tantawy M. A., Belal A., Alnajjar R., Eldehna W. M., Al-Karmalawy A. A. (2022). Bioorg. Chem..

[cit45] Al-Karmalawy A. A., Nafie M. S., Shaldam M. A., Elmaaty A. A., Antar S. A., El-Hamaky A. A., Saleh M. A., Elkamhawy A., Tawfik H. O. (2023). J. Med. Chem..

[cit46] Mahmoud D. B., Bakr M. M., Al-Karmalawy A. A., Moatasim Y., El Taweel A., Mostafa A. (2022). AAPS PharmSciTech.

[cit47] Maestro-Desmond Interoperability Tools, Schrödinger, New York, NY, USA, 2017

[cit48] Ezz Eldin R. R., Saleh M. A., Alotaibi M. H., Alsuair R. K., Alzahrani Y. A., Alshehri F. A., Mohamed A. F., Hafez S. M., Althoqapy A. A., Khirala S. K., Amin M. M., F Y. A., AbdElwahab A. H., Alesawy M. S., Elmaaty A. A., Al-Karmalawy A. A. (2022). J. Enzyme Inhib. Med. Chem..

[cit49] Pires D. E., Blundell T. L., Ascher D. B. (2015). J. Med. Chem..

[cit50] El-Shiekh R. A., Ali D. E., Mandour A. A., Meselhy M. R. (2024). Ind. Crops Prod..

[cit51] Ibrahim R. M., Abdel-Baki P. M., Mohamed O. G., Al-Karmalawy A. A., Tripathi A., El-Shiekh R. A. (2024). Sci. Rep..

[cit52] Kassem A. F., Omar M. A., Temirak A., El-Shiekh R. A., Srour A. M. (2024). Future Med. Chem..

