# Supplementary material for: Discovery of Caralluma-derived pregnane glycosides as potent and selective cholinesterase inhibitors: integrated in silico and in vitro evaluation
Source: RSC Adv. 2026 May 13;16(27):24903–15. doi: 10.1039/d6ra01548d (PMC13169111; doi:10.1039/d6ra01548d)
Supplement: RA-016-D6RA01548D-s001 [file RA-016-D6RA01548D-s001.pdf]

Supplementary Information (SI) for RSC Advances.
This journal is © The Royal Society of Chemistry 2026

Discovery of *Caralluma*-Derived Pregnane Glycosides as Potent and Selective Cholinesterase Inhibitors: Integrated *In Silico* and *In Vitro* Evaluation

Ahmed A. Al-Karmalawy[1,*], Mohamed Ibrahim Attia[2], Radwan Alnajjar[3], Riham A. El-Shiekh[4], Arwa Omar Al Khatib[5], Tarek A. Yousef[2,*], Essam Abdel-Sattar[4]

[1] Department of Pharmaceutical Chemistry, Faculty of Pharmacy, Horus University-Egypt, New Damietta 34518, Egypt.

[2] College of Science, Chemistry Department, Imam Mohammad Ibn Saud Islamic University (IMSIU), Riyadh 11623, Saudi Arabia.

[3] Department of Chemistry, Faculty of Science, University of Benghazi, Benghazi, Libya.

[4] Pharmacognosy Department, Faculty of Pharmacy, Cairo University, Kasr El-Aini Street, 11562 Cairo, Egypt.

[5] Faculty of Pharmacy, Al-Ahliyya Amman University, Amman, Jordan.

*** Corresponding authors**:

Ahmed A. Al-Karmalawy: Email: akarmalawy@horus.edu.eg

Tarek A. Yousef: Email: tayousef@imamu.edu.sa

Spectral Data

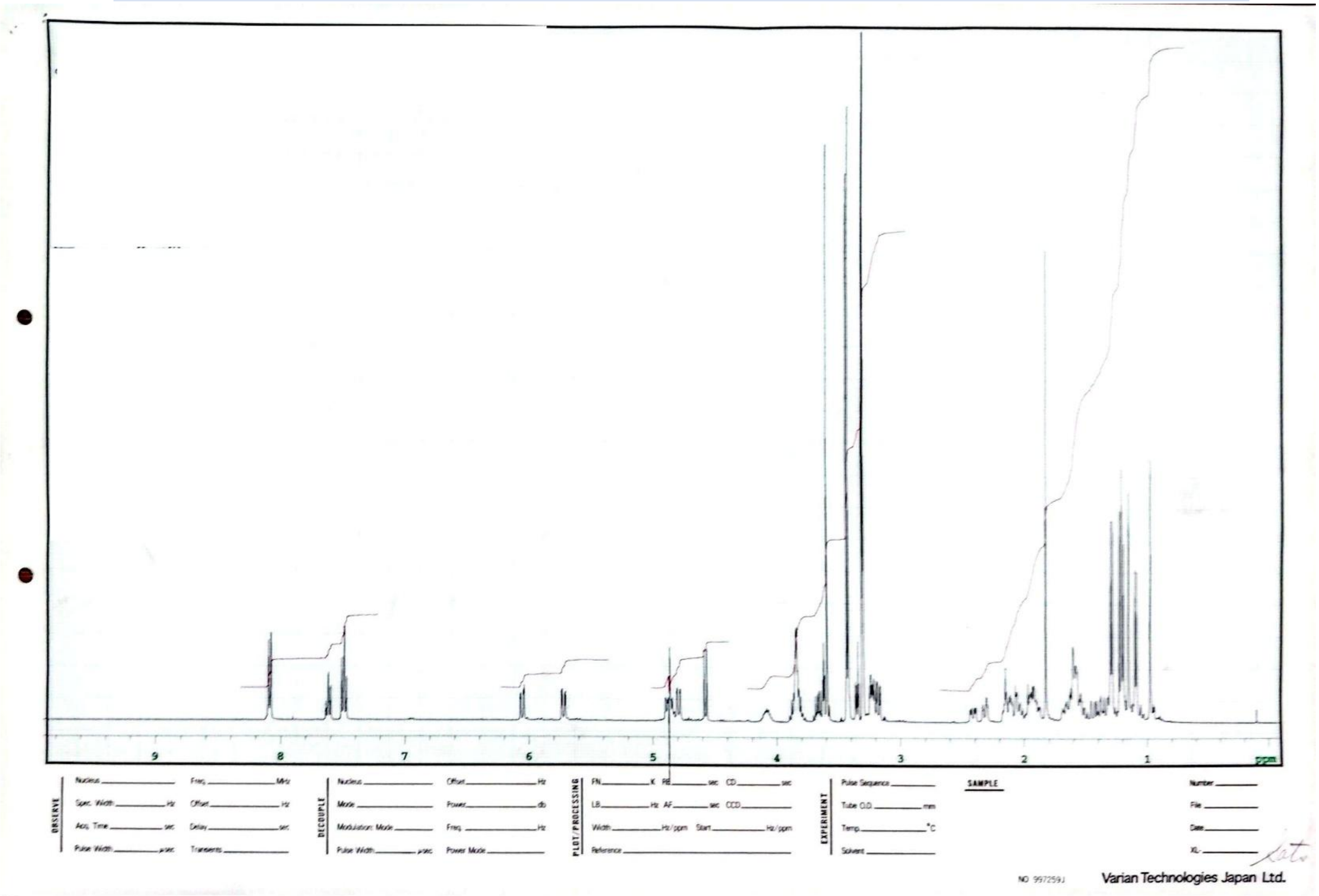

CS Scanned with CamScanner

Fig. S1. ^{1}HNMR of Caratuberside E (10).

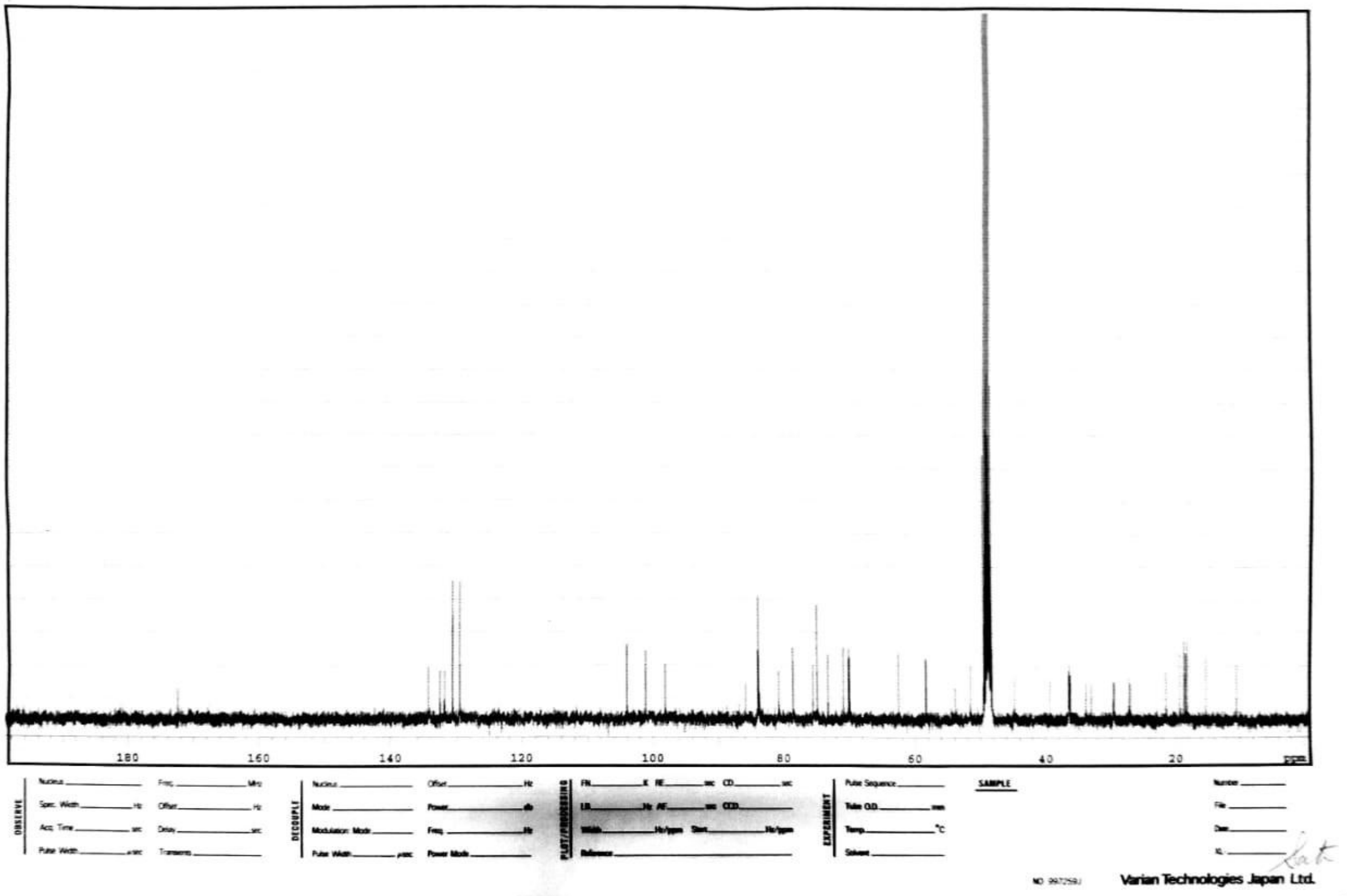

Fig. S2. ^{13}CNMR of Caratuberside E (10).

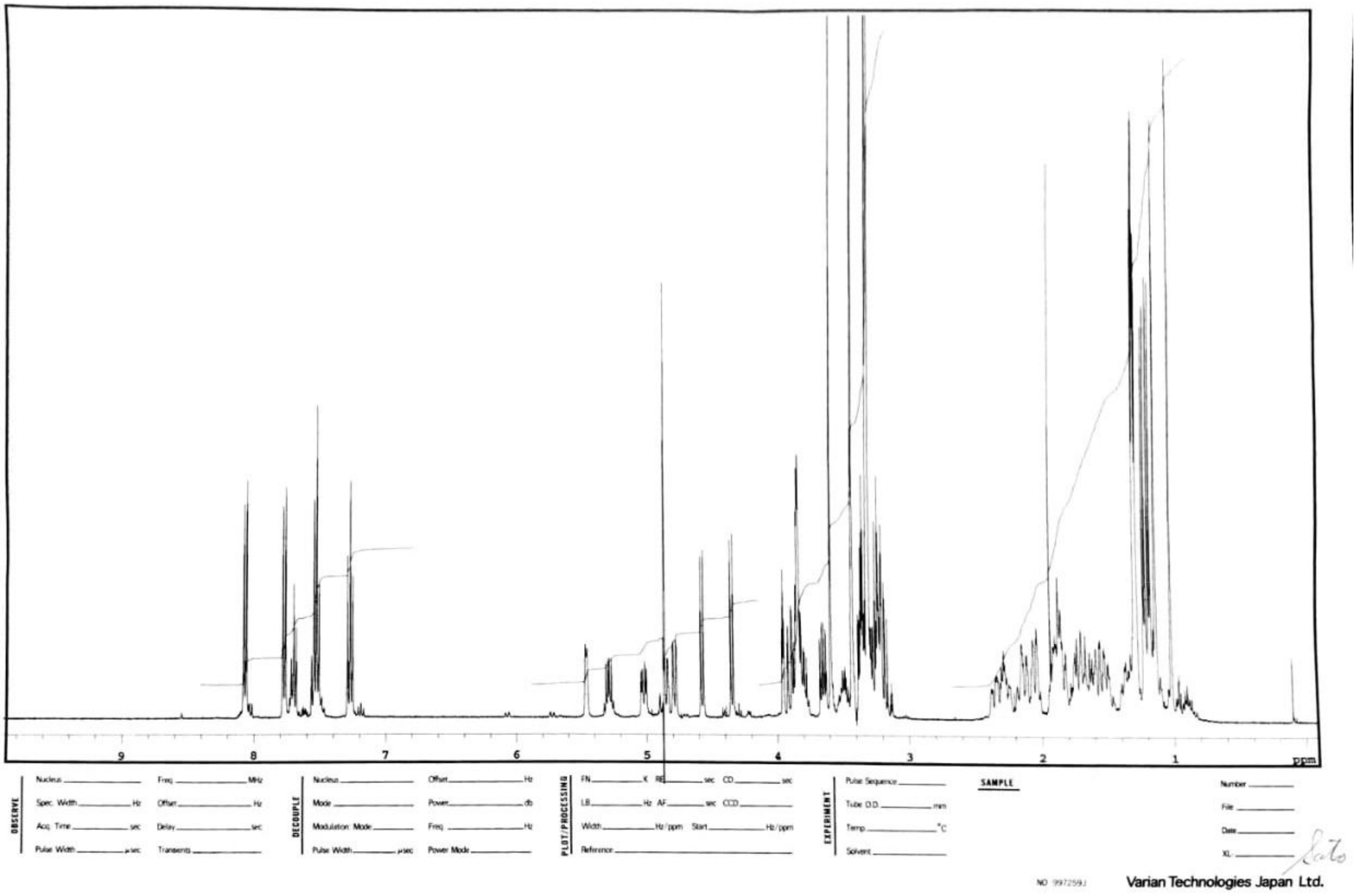

Fig. S3. ^{1}HNMR of Caratuberside G (11).

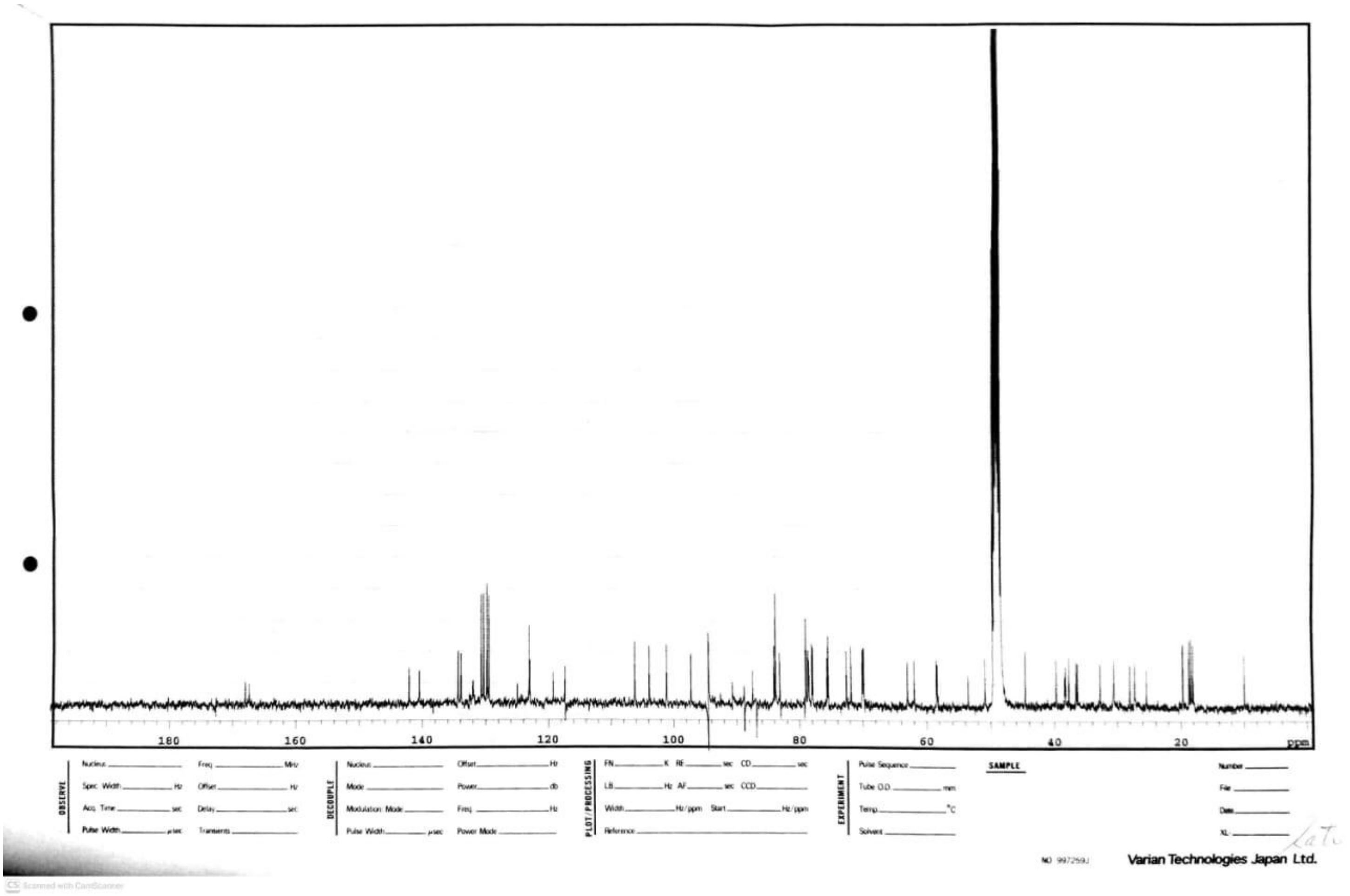

Fig. S4. ^{13}CNMR of Caratuberside G (11).

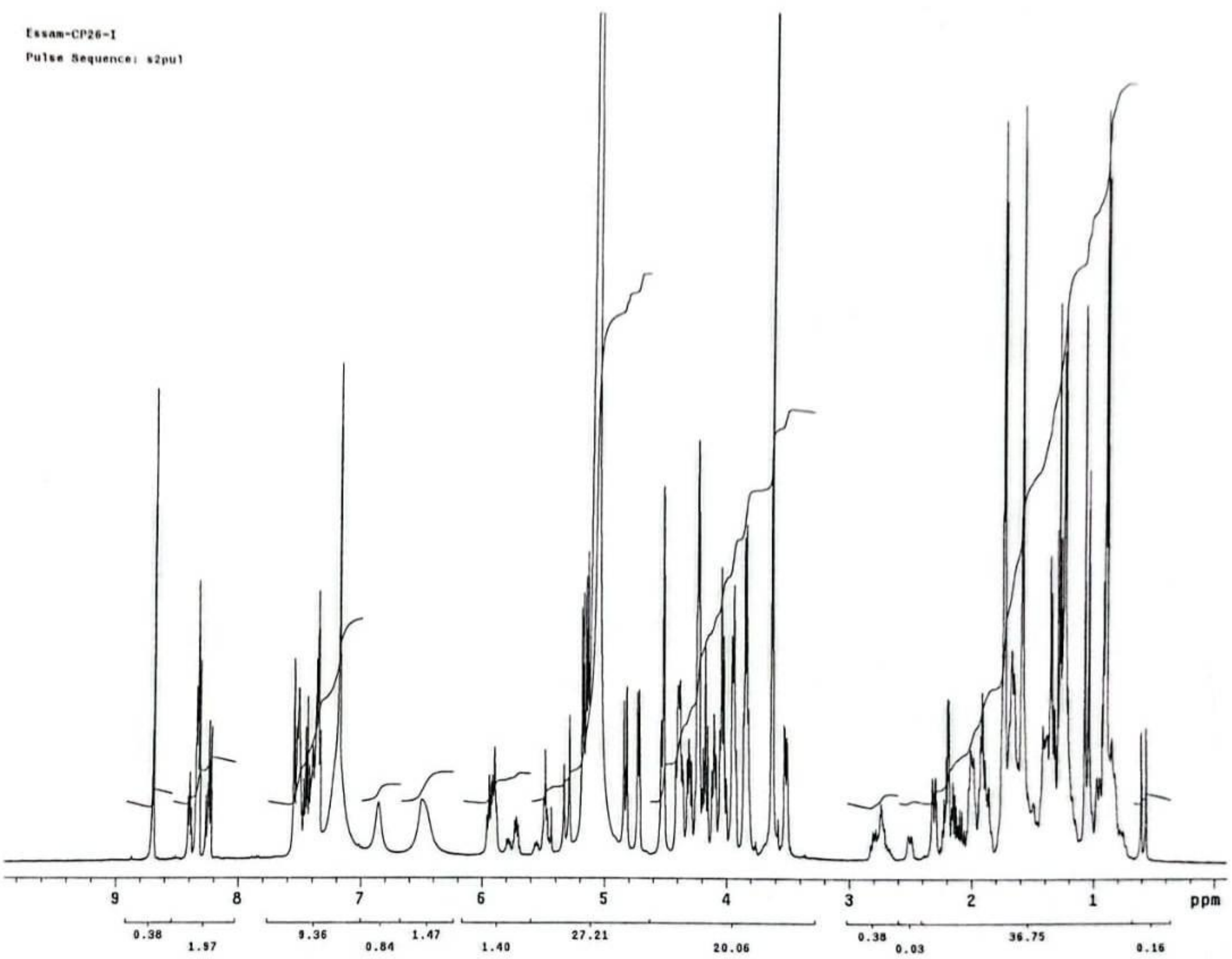

Fig. S5. ^{1}HNMR of penicilloside C (15).

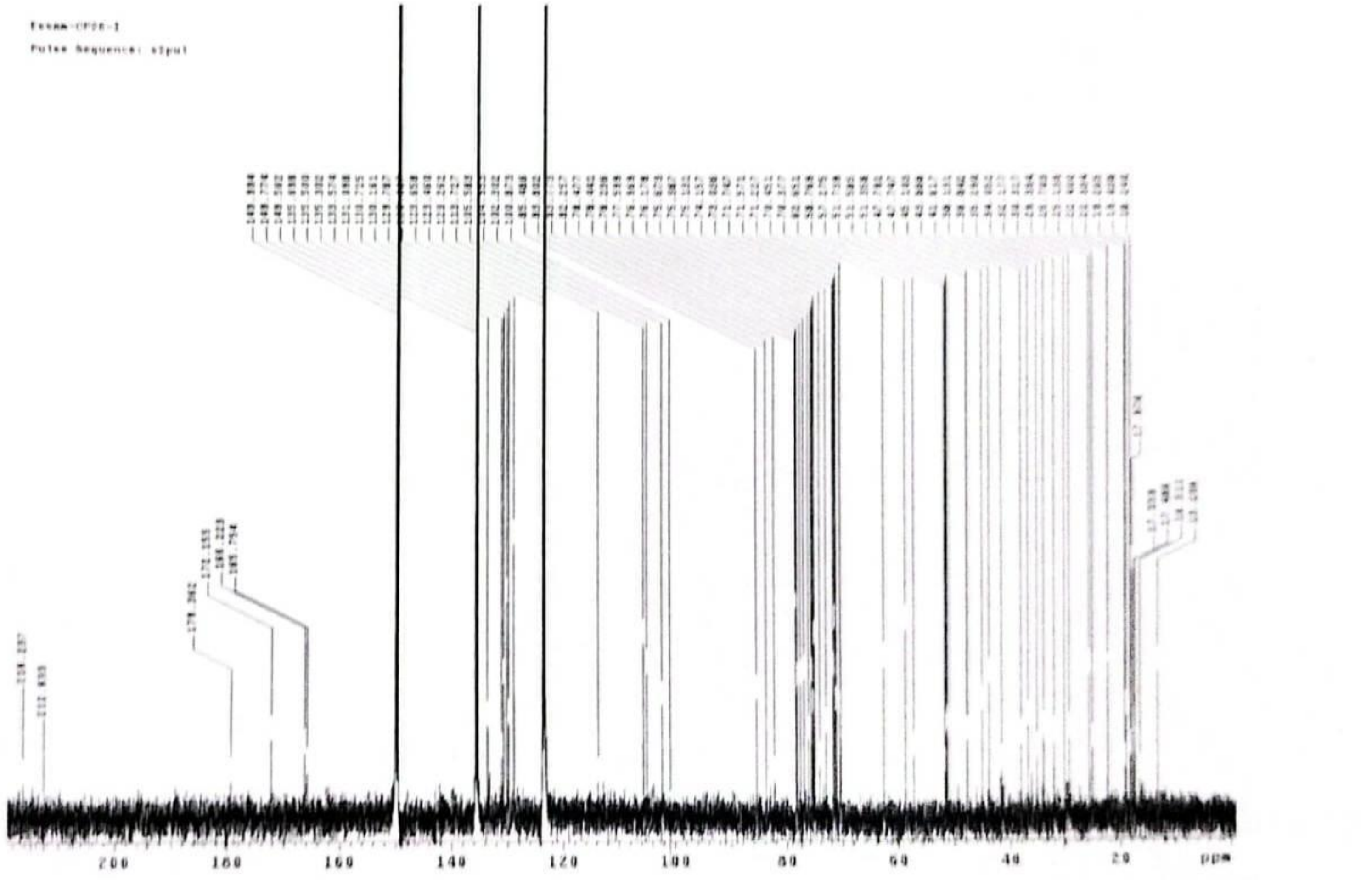

Fig. S6. ^{13}CNMR of penicilloside C (15).

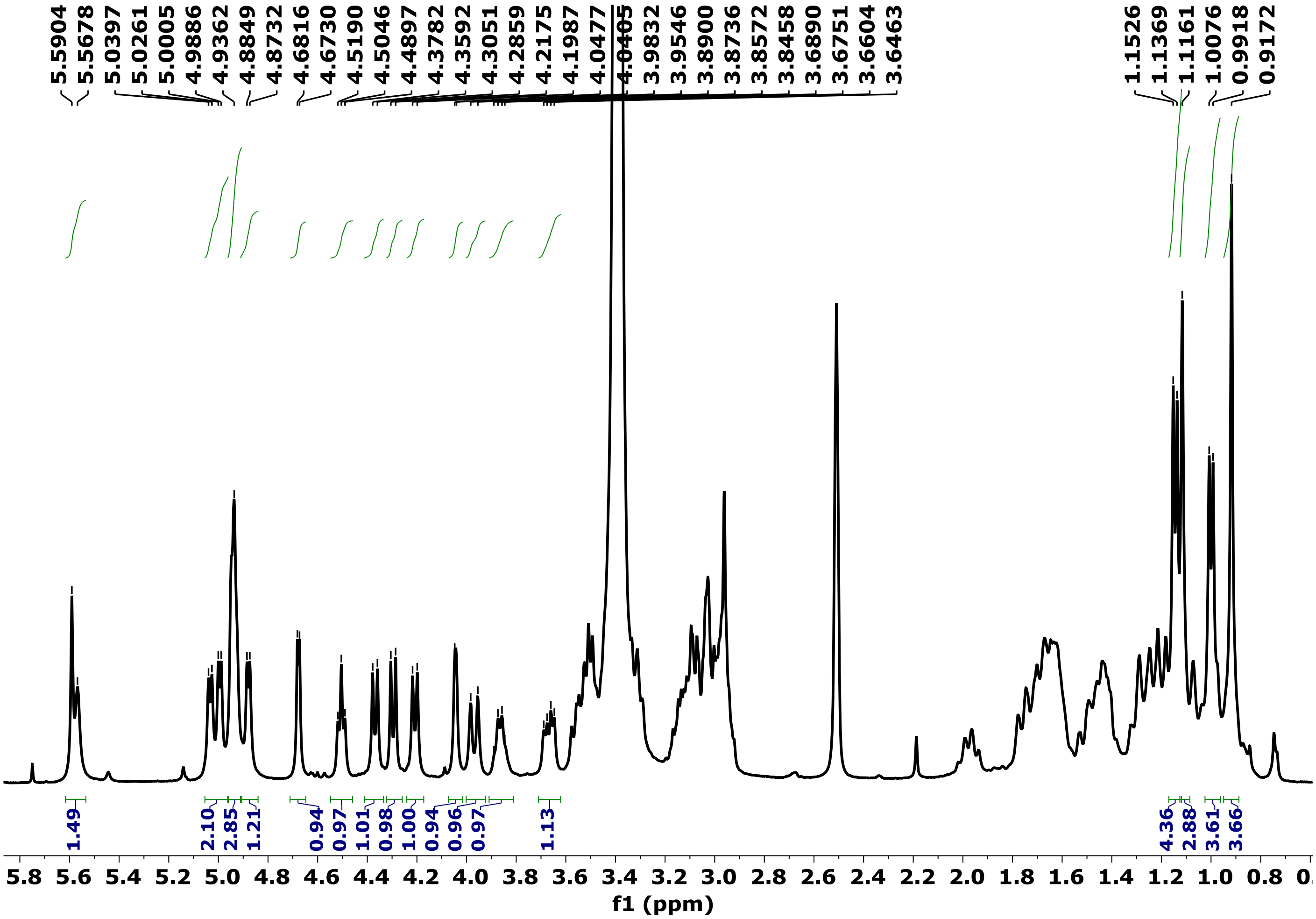

Fig. S7. ^{1}**HNMR of awdelioside B (25)**.

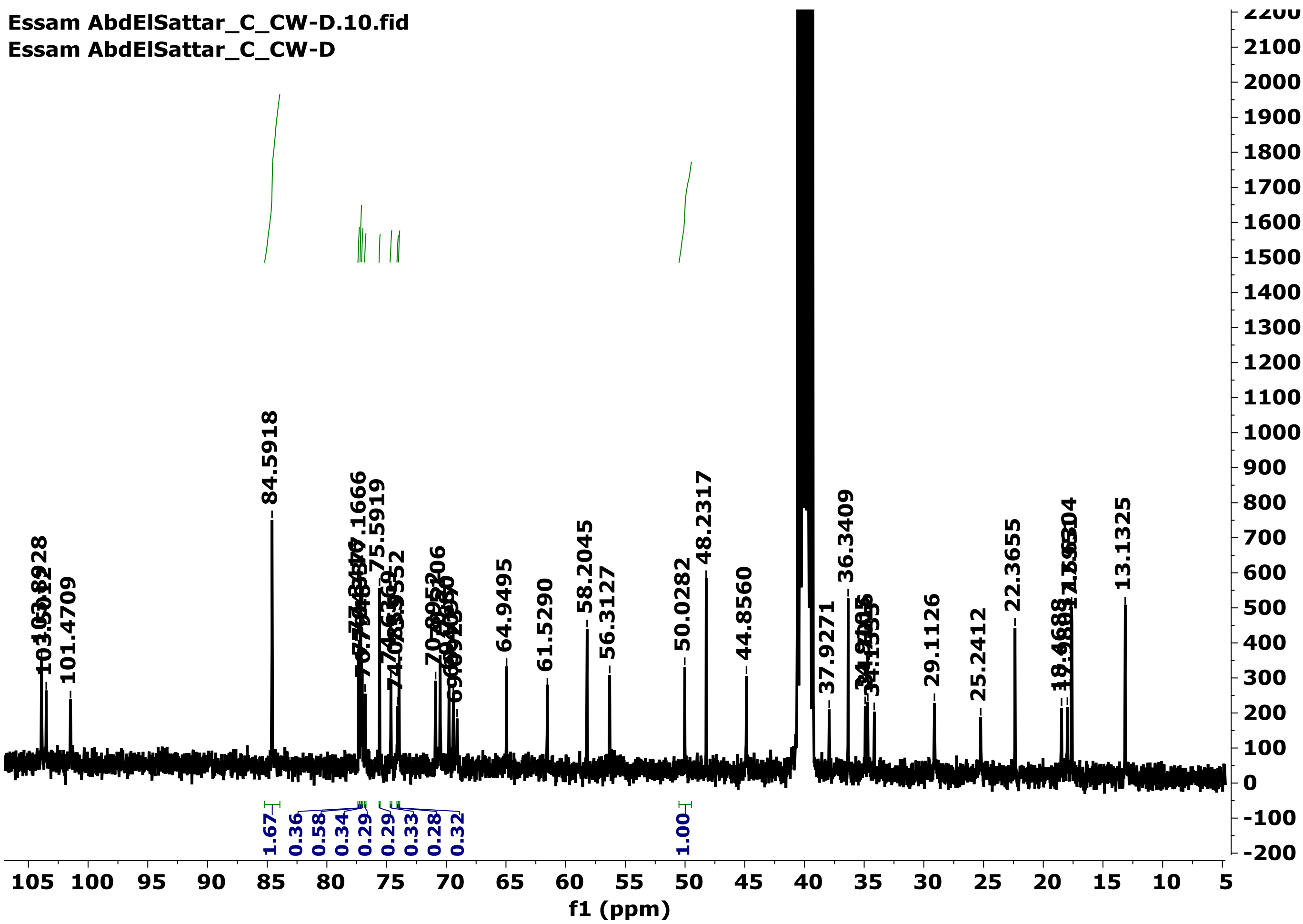

Fig. S8. ^{13}**CNMR of awdelioside B (25).**

Molecular Docking Validation

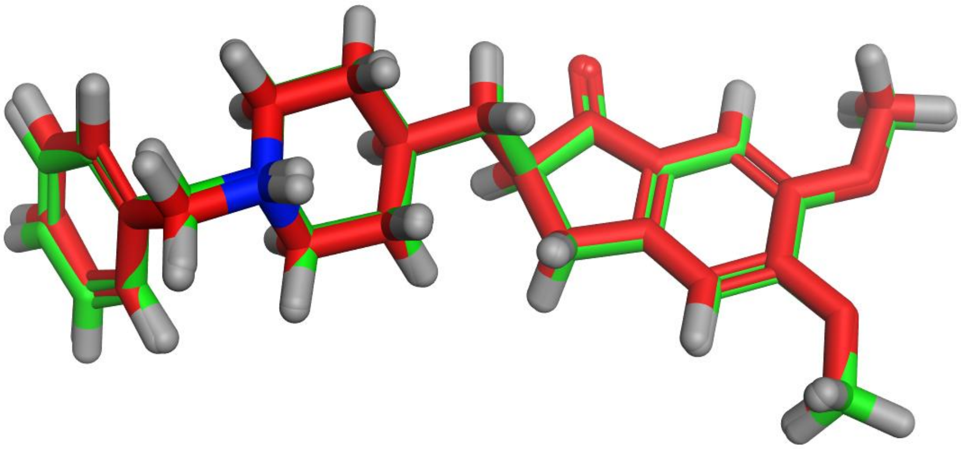

A

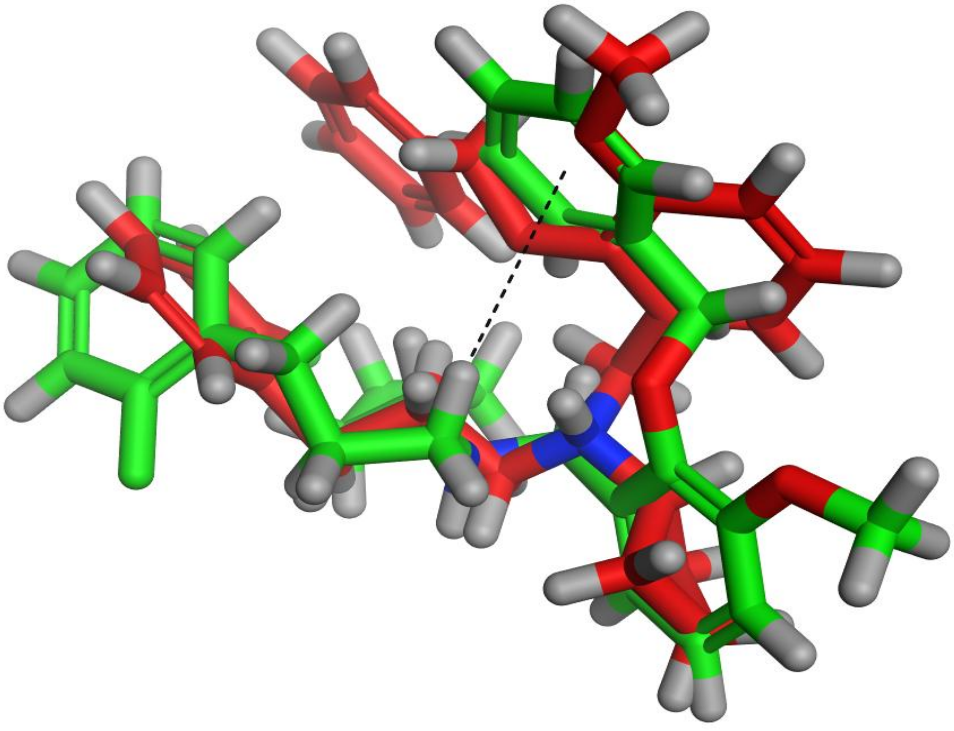

B

Fig. S9. Superimposition of the docked co-crystal (green) over the native co-crystal (red) for (A) AChE (PDB ID: 4EY7 and RMSD = 0.13 Å) and (B) BuChE (PDB ID: 8CGO and RMSD = 1.32 Å).

Molecular Dynamics Simulation Validation

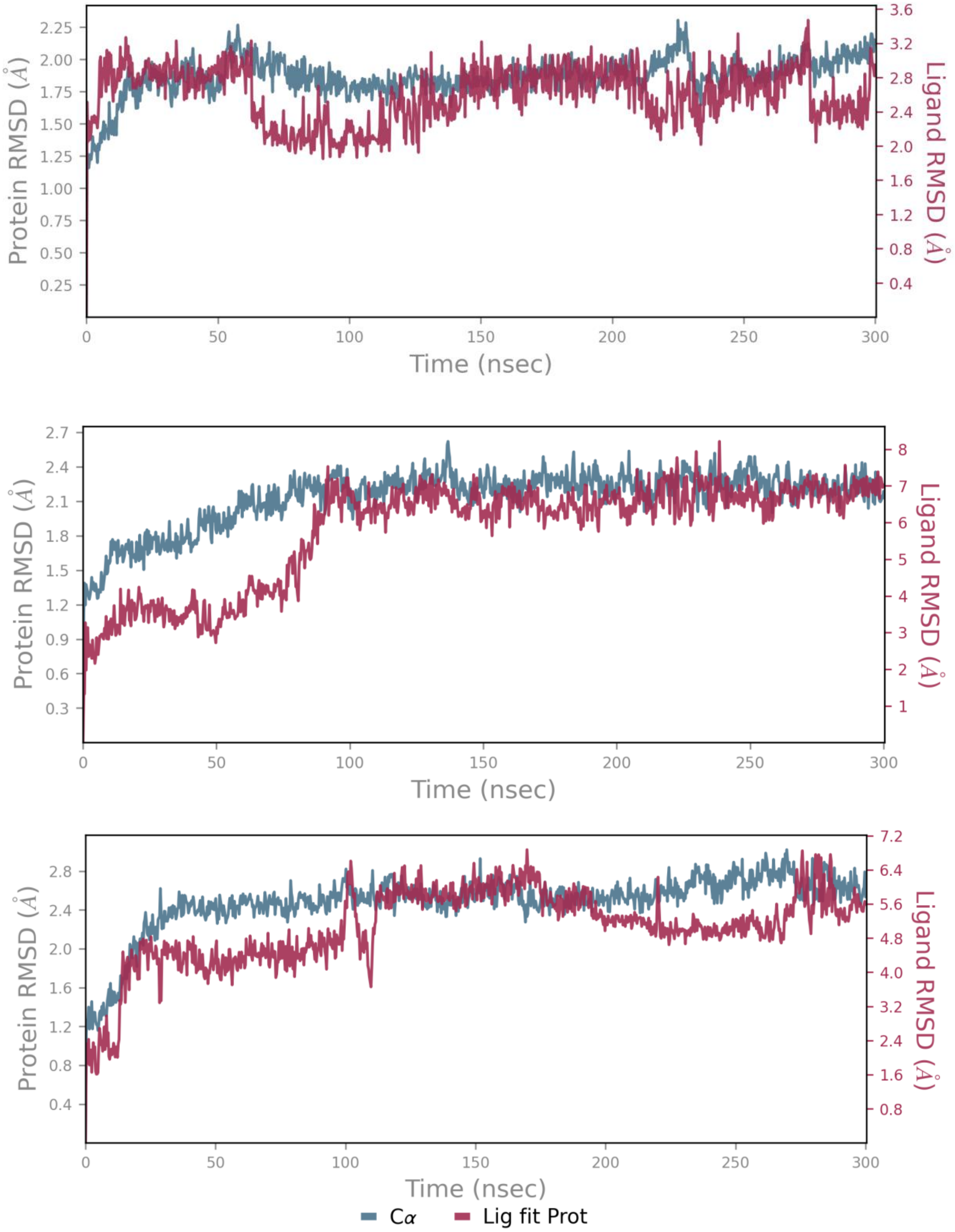

Fig. S10. Three runs were conducted on Caratuberside E for 300 ns as a proof-of-concept.

Molecular Dynamics Simulation Validation

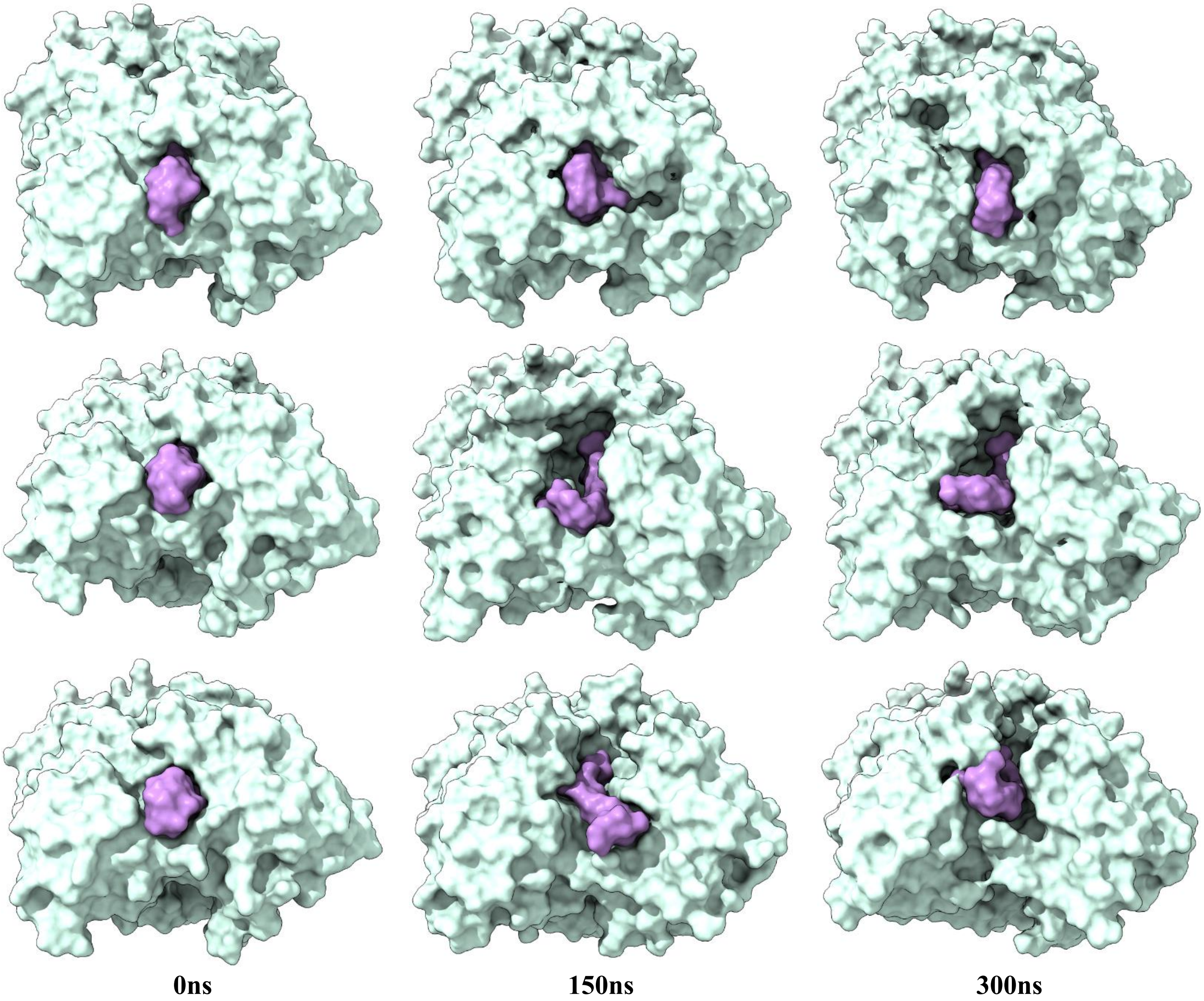

Fig. S11. Snapshots of the triplicate run at 0, 150, and 300 ns.

In Silico Studies

SI1. Molecular dynamics simulation

The molecular dynamics simulations were carried out using the Desmond simulation package of Schrödinger LLC.[1-3] The NPT ensemble with the temperature 300 K and a pressure of 1.01 bar was applied in all runs. The simulation length was 200 ns with a relaxation time of 1 ps. The OPLS4 force field parameters were used in all simulations.[4] The cutoff radius in Coulomb interactions was 9.0 Å. The orthorhombic periodic box boundaries were set 10 Å away from the protein atoms. The water molecules were explicitly described using the transferable intermolecular potential with the three-point (TIP3P) model.[5] Salt concentration was set to 0.15 M NaCl and was built using the System Builder utility of Desmond. The Martyna−Tuckerman−Klein chain coupling scheme with a coupling constant of 2.0 ps was used for the pressure control, and the Nosé−Hoover chain coupling scheme for the temperature control.[6, 7] Nonbonded forces were calculated using a RESPA integrator where the short-range forces were updated every step, and the long-range forces were updated every three steps. The trajectories were saved at 300 ps intervals for analysis. The behavior and interactions between the ligands and protein were analyzed using the Simulation Interaction Diagram tool implemented in the Desmond MD package. The stability of MD simulations was monitored by looking at the RMSD of the ligand and protein atom positions as a function of simulation time.

SI2. MD trajectory analysis and prime MM-GBSA calculations

The simulation interactions diagram panel of the Maestro software was used to monitor the contribution to the ligand-protein stability. The molecular mechanics generalized born/solvent accessibility (MM – GBSA) was performed to calculate the ligand binding free energies and ligand strain energies for docked compounds over the last 50 ns with thermal_mmgbsa.py python script provided by Schrodinger which takes a Desmond trajectory file, splits it into individual snapshots, runs the MM-GBSA calculations on each frame, and outputs the average computed binding energy.

References

1. K. J. Bowers, D. E. Chow, H. Xu, R. O. Dror, M. P. Eastwood, B. A. Gregersen, J. L. Klepeis, I. Kolossvary, M. A. Moraes, F. D. Sacerdoti, J. K. Salmon, Y. Shan and D. E. Shaw, 2006.
2. M. H. El-Shershaby, A. Ghiaty, A. H. Bayoumi, A. A. Al-Karmalawy, E. M. Husseiny, M. S. El-Zoghbi and H. S. Abulkhair, *Bioorganic & Medicinal Chemistry*, 2021, **42**, 116266.
3. D. E. S. Research, *Journal*, 2021.
4. E. Harder, W. Damm, J. Maple, C. Wu, M. Reboul, J. Y. Xiang, L. Wang, D. Lupyan, M. K. Dahlgren, J. L. Knight, J. W. Kaus, D. S. Cerutti, G. Krilov, W. L. Jorgensen, R. Abel and R. A. Friesner, *Journal of Chemical Theory and Computation*, 2016, **12**, 281-296.
5. W. L. Jorgensen, J. Chandrasekhar, J. D. Madura, R. W. Impey and M. L. Klein, *Journal of Chemical Physics*, 1983, **79**, 926-935.
6. G. J. Martyna, M. L. Klein and M. Tuckerman, *Journal of Chemical Physics*, 1992, **97**, 2635-2643.
7. G. J. Martyna, D. J. Tobias and M. L. Klein, *Journal of Chemical Physics*, 1994, **101**, 4177-4189.
